# Technologies for Targeted RNA Degradation and Induced RNA Decay

**DOI:** 10.1021/acs.chemrev.4c00472

**Published:** 2024-11-05

**Authors:** Sigitas Mikutis, Gonçalo J. L. Bernardes

**Affiliations:** Yusuf Hamied Department of Chemistry, University of Cambridge, Lensfield Road, Cambridge CB2 1EW, U.K.

## Abstract

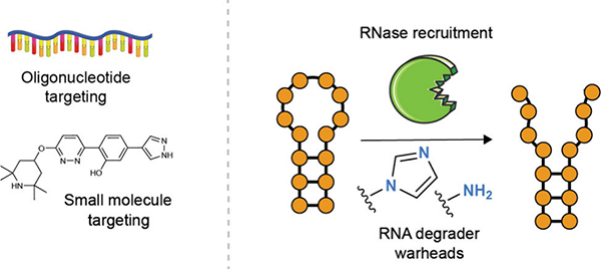

The vast majority of the human genome codes for RNA, but RNA-targeting therapeutics account for a small fraction of approved drugs. As such, there is great incentive to improve old and develop new approaches to RNA targeting. For many RNA targeting modalities, just binding is not sufficient to exert a therapeutic effect; thus, targeted RNA degradation and induced decay emerged as powerful approaches with a pronounced biological effect. This review covers the origins and advanced use cases of targeted RNA degrader technologies grouped by the nature of the targeting modality as well as by the mode of degradation. It covers both well-established methods and clinically successful platforms such as RNA interference, as well as emerging approaches such as recruitment of RNA quality control machinery, CRISPR, and direct targeted RNA degradation. We also share our thoughts on the biggest hurdles in this field, as well as possible ways to overcome them.

## 1 Introduction

RNA plays a very diverse set of roles in biology, perhaps more so than any other biomolecule. Unlike the structurally related DNA, RNA synthesis and degradation are extremely dynamic, with half-lives ranging from tens of seconds to tens of hours for mRNAs^1,2^ to several days for rRNAs^3,4^ to even longer timeframes for protein-bound miRNAs. To maintain this dynamic behavior, cells have evolved numerous RNA degradation mechanisms, some with precise control over the target and location of degradation and some that are rather promiscuous. Misregulation of these mechanisms can result in disease. To give some examples, defects in the miRNA system of genes are associated with oncologic conditions,^5,6^ whereas defects in nucleases which are part of the innate cellular immunity will make cells more susceptible to viral infections.^7^ Many of these RNA regulation systems have been adapted or served as inspiration to develop tools that degrade the desired RNAs at will, both to advance the understanding of the functions of targeted RNAs as well as to develop new therapeutic methods.

**Figure 1:**
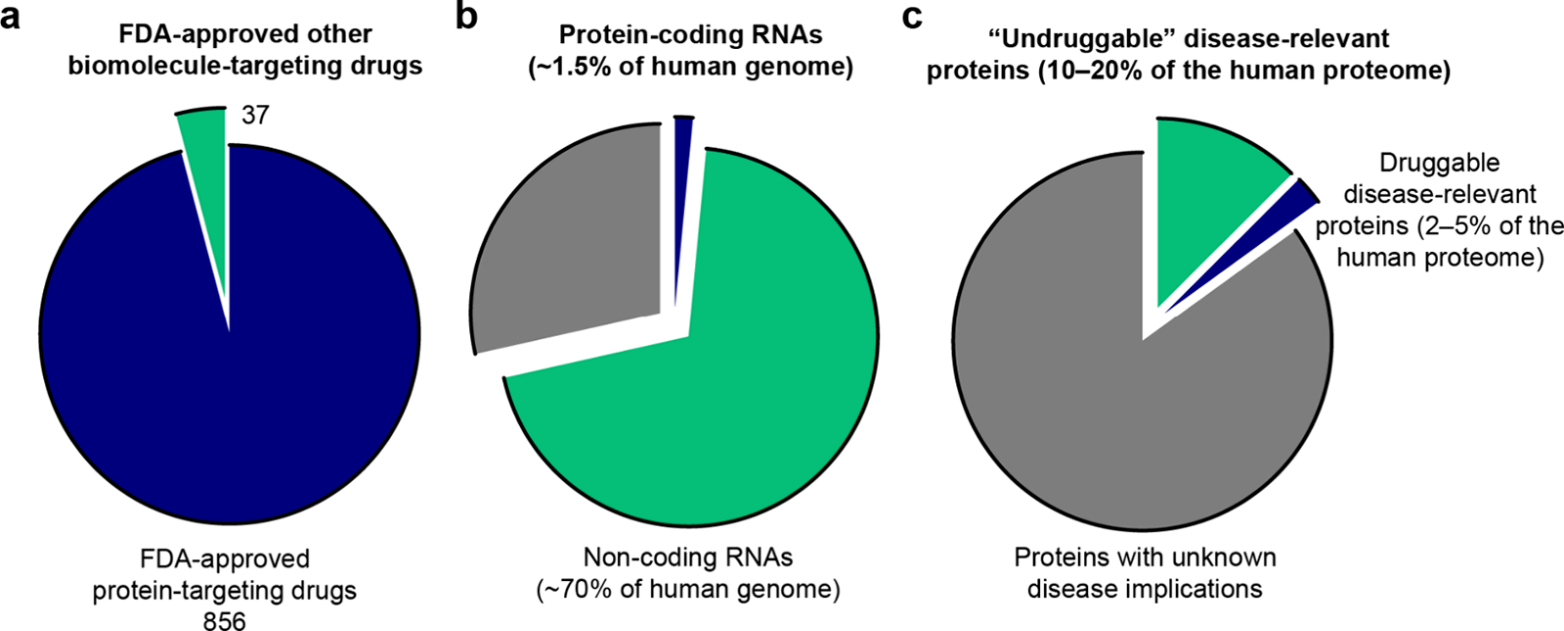
RNA-targeting approaches can dramatically expand the space of druggable biomolecules. (a) The vast majority of FDA-approved drug targets are proteins. (b) Only a small fraction of the human genome codes for proteins, but the majority codes for RNA. (c) Only a fraction of the disease-relevant human proteome is druggable via traditional medicinal chemistry approaches, but the druggable protein space could be expanded by targeting them at the RNA level.

But why do we want to therapeutically target RNA in the first place? Protein targeting approaches remain the central pillar of modern therapeutics development—by 2019, FDA-approved drugs were targeting 856 proteins and only 37 other biomolecules (Figure 1a).^8^ However, just 1.5% of the human genome codes for proteins, and the majority of those 1.5% do not have well-defined binding surfaces, which hinders drug development (e.g., transcription factors are key players in numerous diseases, but most are considered “undruggable”) (Figure 1b, c).^9−11^ When it comes to RNA, around 70% of the human genome is transcribed—an enormous jump from the space of druggable proteins.^12^ Furthermore, the ability to selectively degrade RNAs at will can also be utilized to modulate the protein space—e.g., mRNAs encoding either druggable or “undruggable” proteins can be degraded to deplete them. As such, there is a huge incentive to develop new tools to target and degrade RNA, as the newly unlocked biomolecular target space will undoubtedly present new avenues to cure diseases that are yet untreatable.

**Figure 2:**
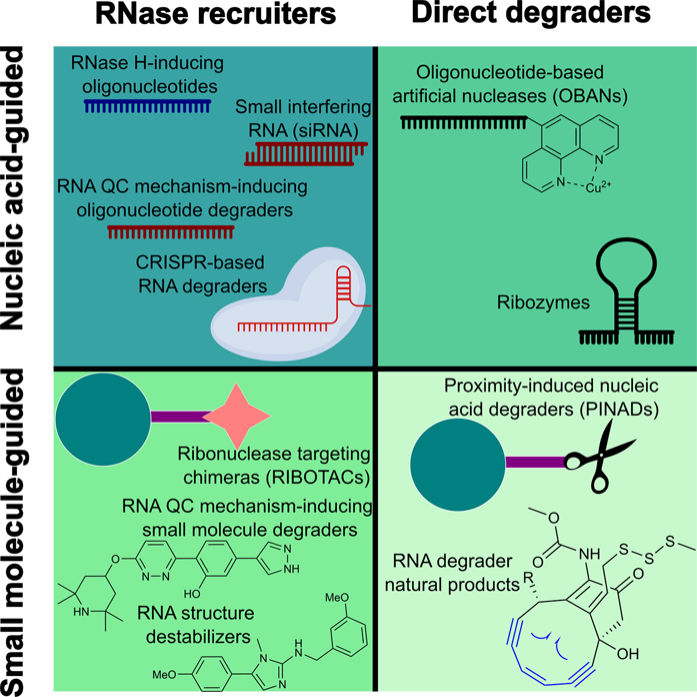
Matrix of targeted RNA degradation methods described in this review.

These tools to degrade RNA in a targeted manner are the focus of this review (Table 1, Figure 2). This review divides them into two groups, based on the targeting method, with an emphasis on translational approaches. The first, nucleic-acid-guided methods, covers tools where the targeting is achieved via guiding nucleic acids, targeting RNA in a sequence-dependent manner. The second is small-molecule-guided methods, which typically target RNA in a structure-dependent manner. While distinct in the targeting strategy and the sphere of applicability, they share much in degradation approaches, and in many cases, nucleic-acid-based methods serve as inspiration for small molecule approaches. Due to the crosstalk between these strategies, it is beneficial to look at them together; thus, this review will contrast various aspects of the two approaches. The most advanced targeted RNA degradation methods rely on ribonuclease recruitment; thus, they will be covered first, followed by emerging direct RNA degradation methods. The description of each RNA degrading tool will be preceded by an overview of natural systems which served as the inspiration, followed by the description of the tool itself and the most advanced use cases as well as limitations and, where applicable, what may be lacking for the method to find success in the clinic. Key chemical and structural features that enable RNA targeting and degradation are described for each tool. A particular focus will be given to therapeutic applications for each of the methods.

**Table 1:** Targeted RNA Degradation Approaches Discussed in This Review

Targeted RNA degradation method	Targeting strategy	Degradation strategy	The most advanced use case
RNase H-inducing oligonucleotides	Guide oligonucleotides	Recruitment of ribonuclease H	Multiple approved therapeutics
RNA interference (siRNA)	Guide oligonucleotides	Recruitment of ribonuclease AGO2	Multiple approved therapeutics
CRISPR-based methods	Guide oligonucleotides	Recruitment of Cas ribonucleases	Therapeutic application in animal models, clinically tested diagnostics
RNA QC mechanism-induced degradation	Guide oligonucleotides or small molecules	Recruitment of RNA quality control ribonucleases	Small molecules in clinical trials
Direct RNA degraders	Guide oligonucleotides or small molecules	Degradation through RNA degrader warheads	Therapeutic application in animal models
Ribozymes	Guide RNA sequence	Degradation through RNA catalysis	Clinical trials
RNase L-inducers	Small molecules	Recruitment of ribonuclease L	Therapeutic application in animal models
Degrader natural products	Guide oligonucleotides or small molecules	Degradation through natural products	Therapeutic application in animal models
RNA structure disruptors	Small molecules	Disruption of RNA degradation-preventing structures	Therapeutic application in cell culture

## 2 Nucleic-Acid-Guided Methods

Targeting RNA with oligonucleotides is perhaps the oldest and the most well-explored method to modulate both stability and function of RNA species. Antisense oligonucleotide technology was first described by the team of Paul Zamecnik, when they reported that a DNA oligonucleotide complementary to a tract in the Rous Sarcoma virus 35S rRNA subunit inhibits viral replication.^13^ The authors of the initial publication suggested that the oligonucleotide acts as a steric blocker, which binds RNA and prevents interactions with native partners. However, the reality turned out to be more complicated than that—the resulting RNA:DNA hybrid could be recognized and degraded by ribonuclease H. Thus, it turned out that DNA oligonucleotides can function not merely as stoichiometric RNA binders but also as catalytic RNA degraders.^14−16^ Other nucleic-acid-guided degrader technologies were to follow—siRNAs are already well established and have made numerous inroads into the clinic, and CRISPR-Cas13 and various class 1 CRISPR systems have already been made into tools reported to be advantageous over existing methods.^17−19^ Antisense oligonucleotides are also being harnessed to utilize various RNA quality control mechanisms for selective degradation.

Before moving on to more granular discussion, we will define the key features, scope, and limitations of nucleic-acid-guided RNA degradation tools. Depending on the biological mechanism used, these guides are composed or either DNA or RNA typically between 10 and 30 oligonucleotides long, and are typically heavily modified as nonmodified oligonucleotides get rapidly degraded inside and outside cells.^20^ These oligonucleotides target a particular sequence by forming base pairs with it. The oligonucleotide needs to be able to displace native binders if the targeted sequence has any—this can be facilitated via chemical modifications which increase the RNA binding strength. Nucleic-acid-based therapeutics have some inherent drawbacks—the uptake of naked nucleic acids is very poor; thus, to be useful, they must either be conjugated to a targeting ligand or used in conjunction with a delivery vehicle; their production cost can be high, and they are not typically orally bioavailable.^21^ Oligonucleotide therapeutics often induce adverse reactions and toxicity, which has resulted in withdrawals of FDA approval.^22^ With these features and limitations in mind, let us explore the mechanism of RNase H-inducing oligonucleotides and their journey to becoming one of the most successful classes of RNA-targeting therapeutics.

### 2.1 RNase H-Inducing Oligonucleotides

**Figure 3:**
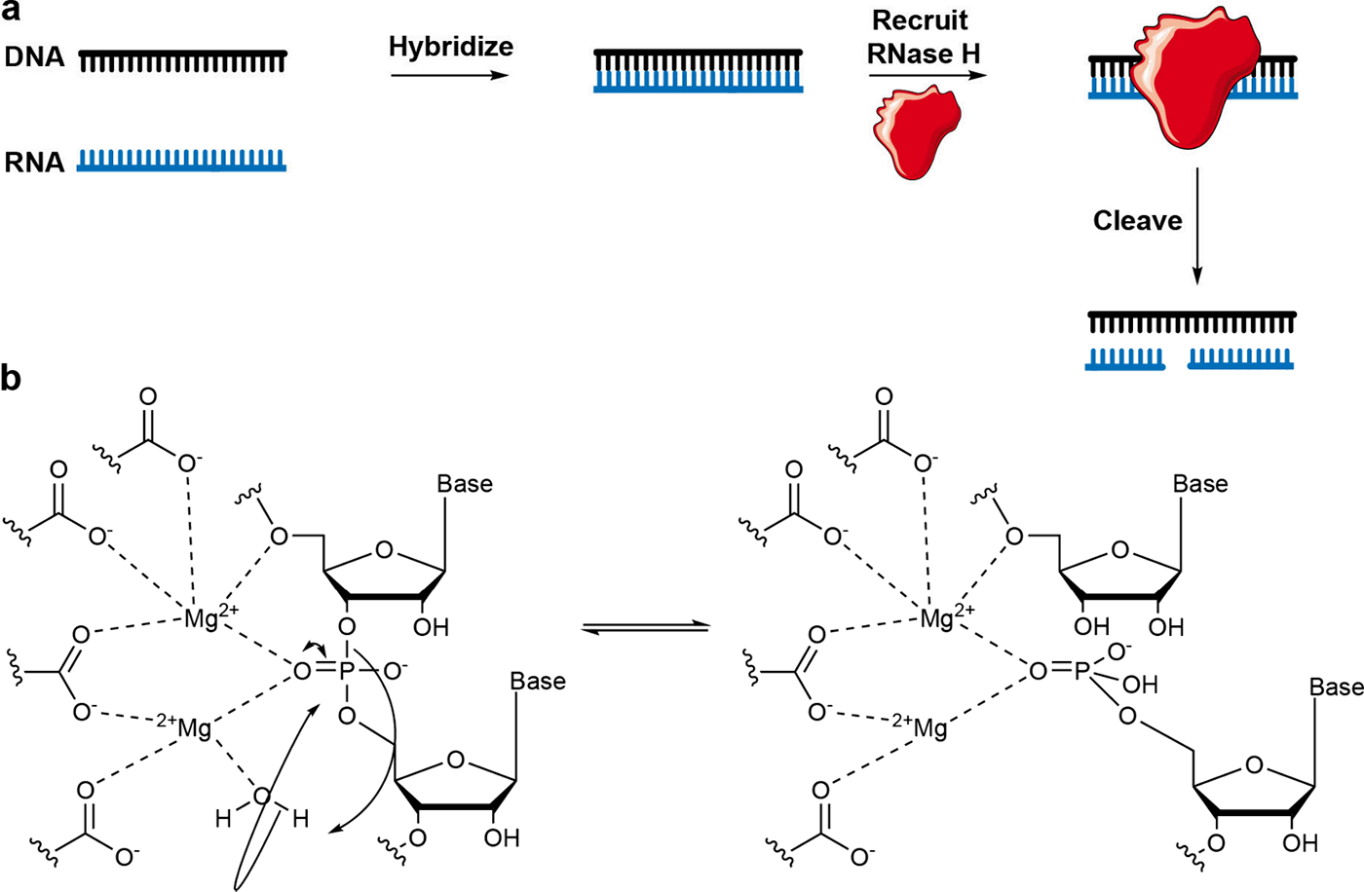
Mechanism of RNase H-induced RNA cleavage. (a) RNase H cleaves RNA in an RNA:DNA duplex. (b) Simplified molecular mechanism of RNase H-induced cleavage of the phosphodiester bond.

RNases H are ribonucleases that bind DNA:RNA duplexes and cut the RNA strand via a hydrolytic mechanism. Although these enzymes can also bind DNA:DNA and RNA:RNA duplexes, they will only cleave DNA:RNA hybrids (Figure 3a).^23−26^ This cleavage selectivity arises from the differential structure of the DNA:RNA helix—the active site of RNase H interacts with the minor groove of the duplex, and only the DNA:RNA hybrid has the correct geometry to position a ribonucleotide for cleavage, due to the shape of the helix and the puckering of ribose.^27,28^ The active site of RNase H contains aspartate and glutamate group amino acid side chains which act as a matrix for divalent metal ion (preferably Mg^2+^ or Mn^2+^) binding (Figure 3b).^29−31^ Metal ions bind the phosphodiester to be cleaved; furthermore, they bind a water molecule, which is activated as a nucleophile and cleaves the bound phosphodiester. The cleavage results in 5′-phosphate and 3′-hydroxyl ends.^32^ The two metal ion cleavage mechanism of RNase H is shared with many DNases; thus, stringent structural requirements are in place to prevent cleavage of DNA.^33^

The RNase H-induced RNA cleavage in DNA:RNA duplexes is vital for DNA replication across all of the domains of life. In eukaryotes, RNase H1 is needed for initiation of mitochondrial DNA replication (RITOLS model).^34−36^ It removes RNA primers on the growing DNA chain as the failure to do so results in an RNA:DNA duplex, which blocks the activity of mitochondrial DNA polymerase in subsequent replication cycles.^37,38^ It was demonstrated that knocking down the *RNASEH1* gene induces embryo development defects in mice and cell replication defects in humans, due to failure to produce mitochondrial DNA.^39^ RNase H is also key for replication of retroviruses where it is found as a domain of the reverse transcriptase holoenzyme complex.^30,40,41^ Retroviral RNase H has also been explored as a therapeutic target for antiviral drug development, with many of the developed compounds binding the two magnesium atoms in its active site.^42−44^

As mentioned in the introduction of this section, recruitment of RNase H (in particular, the human RNase H1, encoded by the *RNASEH1* gene) was one the first targeted RNA degradation methods harnessed for therapeutic purposes, in the form of RNase H inducing oligonucleotides. They take an advantage of the rather unique ability of RNase H to cleave DNA:RNA hybrids and typically are modified DNA oligonucleotides with a sequence complementary to a segment of the targeted RNA species, usually up to 25 nucleotides long.^45^ RNase H-inducing oligonucleotides form an DNA:RNA duplex with the targeted RNA species, which in a cellular environment leads to recruitment of RNase H and, subsequently, cleavage of the targeted RNA.^46,47^

One of the key challenges in the development of RNase H-inducing ASOs is metabolic stability—endogenous DNases will rapidly degrade DNA oligonucleotides, with half-lives as short as several minutes.^48,49^ The way to circumvent this problem is to use modified deoxyribonucleotides, which hamper recognition by DNases. However, RNase H has exquisite selectivity for DNA:RNA duplex geometry, thus any modifications that disrupt this structure also abolish the mechanism of degradation.^26^ As such, a fine balance must be struck—too little modifications, and the oligonucleotide will get degraded too quickly to exert an effect; too many modifications, and the oligonucleotide will not be able to recruit the RNase H. Two principles in the design of RNase H-inducing oligonucleotides are typically followed. First, use of modifications that do not hinder the recognition by RNase H.^50^ Second, design of gapmers—oligonucleotides consisting of heavily modified flanks which confer exonuclease protection and lightly modified center which can still be recognized by RNase H. Other reviews offer a thorough coverage of the modification space in the context of RNase H-inducing oligonucleotides; this review only introduces modifications that led to approved drugs.^50−52^

Perhaps the most widely used modification in oligonucleotides is the phosphorothioate linkage, which replaces the native phosphodiester bonds. First made in the ′60s and synthesis coming into use with automated methods in the ′80s, their success is owed to two key aspects.^53,54^ First, they confer resistance against DNases, with the rate of enzymatic hydrolysis of the phosphorothioate linkage being at least an order of magnitude slower than that of unmodified phosphodiester.^55,50^ Second, they are otherwise similar to phosphodiester bonds—they both are negatively charged, have similar shapes, and are structurally similar enough to be recognized by RNase H.^56,57^ They do have one key difference when compared to native phosphodiesters: the phosphorothioate linkage is chiral. This has consequences for stability, RNA binding, and recognition by RNase H; however, there is no clear consensus on whether stereochemically enriched phosphorothioate oligonucleotides are superior to ones with random stereochemistry.^58−61^ Phosphorothioate oligonucleotides also exhibit differential and typically stronger interactions with proteins compared to phosphodiester analogs.^62^ Oligonucleotides containing a sufficient proportion of phosphorothioate linkages will be covered with plasma proteins when in circulation (protein corona), which can improve the biodistribution to various tissues, promote entry to some cell types and prevent rapid clearance via glomerular filtration characteristic to nonmodified oligonucleotides. This ability to form a protein corona is the reason that RNase-inducing oligonucleotides do not require dedicated delivery. However, interactions of phosphorothioate oligonucleotide with paraspeckle proteins can lead to their localization to the nucleolus, which results in inhibition of rRNA transcription and processing, leading to apoptosis.^63,64^

Another modification present on all approved RNase-inducing antisense oligonucleotides is the 2′-O-(2-methoxyethyl) (2′-MOE) group. As this modification interferes with recognition by RNase H, it is found on the flanks of the gapmer oligonucleotides. Initially designed with the aim of increased RNA binding strength, it was also found to provide exquisite resistance to nucleases compared both to naked DNA as well as 2′-O-Methyl modification.^65,66^ It was discovered that in rat tissues, phosphorothioate gapmers modified with 2′-MOE groups on flanks have 10-fold increased half-lives compared to oligonucleotide with phosphorothioate as the only modification, as well as having lower immunogenicity in mice.^67,68^ Owing to these properties, 2′-MOE modifications as well as phosphorothioate backbones are found in most clinically relevant RNase-H inducing oligonucleotides.^69^

Understanding of both the mechanism of RNase H as well as the role of modification in the design of antisense oligonucleotides have made RNase H-inducing oligonucleotides into a successful class of drugs, with four approved either by the FDA or EMA to date (Table 2).^70−73^ From the design perspective, they are all very similar—all are 20 nucleotides long, three of four have phosphodiester linkages fully replaced with phosphorothioate, whereas in tofersen 15 out of 19 linkages are replaced; all are gapmers, with five flanking nucleotides on both 5′ and 3′ ends bearing the 2′-MOE modification (5–10–5 structure). Three of them have 40% GC content, with mipomersen having 65%.^74^ Notably, all have been developed by Ionis Pharmaceuticals, which has explored various types of antisense oligonucleotides since the late ′80s.

**Table 2:** Approved RNase H-Inducing Oligonucleotide Therapeutics

Name of the oligonucleotide	First approval year	mRNA target	Indication	Administration
Mipomersen (Kynamro)	2013 (retracted)	*APOB100*	Homozygous familial hypercholesterolemia	Subcutaneous, weekly
Inotersen (Tegsedi)	2018	*TTR*	Hereditary transthyretin mediated amyloidosis	Subcutaneous, weekly
Volanesorsen (Waylivra)	2019	*APOC3*	Familial chylomicronemia	Subcutaneous, weekly
Tofersen (Qalsody)	2023	*SOD1*	Amyotrophic lateral sclerosis	Intrathecal, every 2 or 4 weeks

All four approved RNase H-inducing oligonucleotides target mRNA species, which would be extremely difficult to target on a protein level. Mipomersen is FDA-approved for homozygous familial hypercholesterolemia and targets *APOB100*, encoding the apolipoprotein B, a protein playing roles in lipid transport.^74^ Mutations in the *APOB100* can decrease its affinity for low-density lipoprotein (LDL) receptor as well as catabolic rates for associated lipids.^75,76^ This leads to buildup of LDL particles in plasma, which is the underlying cause of the disease. Treatment with mipomersen leads to reduction of *APOB100* mRNA, resulting in decreased levels of apolipoprotein B and LDL particles, which is the mechanism of action for this drug.^77^ However, mipomersen was found to be hepatotoxic, which is why it was discontinued from the market in 2019 and never got an approval from EMA.^22^

Inotersen is used for treatment of hereditary transthyretin-mediated amyloidosis. The molecular basis for this disease is mutations in the *TTR* gene, which encodes transthyretin, a protein responsible for transport of thyroid hormone thyroxin and retinol.^78,79^ This protein is not vital for human health, with mutations which ablate its thyroxin binding ability having no obvious negative impact.^80^ However, some mutations can induce transthyretin aggregation, resulting in formation of neuropathic amyloids, and the resulting tissue damage is the cause of hereditary transthyretin mediated amyloidosis.^81^ This disease can be treated by reducing transthyretin levels, which is inotersen’s mechanism of action—degradation of *TTR* mRNA and reduction of protein and pathogenic amyloid plaques levels.^82^

Volanesorsen is approved by the EMA for treatment of familial chylomicronemia and targets *APOC3* mRNA. It encodes apolipoprotein C3, a key regulator of plasma triglyceride levels.^83^ This protein inhibits lipoprotein lipase (LPL), responsible for metabolism of triglycerides; thus, elevated levels of *APOC3* will lead to buildup of triglycerides in blood, which is the underlying cause of familiar chylomicronaemia. Volanesorsen recruits RNase H1 to reduce the levels of *APOC3* mRNA and the encoded apolipoprotein C3.^84,85^ This leads to increased activity of LPL and lower levels of plasma triglycerides, alleviating the symptoms of the disorder.

Tofersen, approved by the FDA for treatment of amyotrophic lateral sclerosis (ALS) in 2023, targets the *SOD1* mRNA. It encodes superoxide dismutase 1, an enzyme which converts superoxide, a highly reactive oxidant, into less reactive peroxide and dioxygen; thus, it is one of the lines of defense against reactive oxygen species and oxidative stress.^86^ Mutations within this gene can result in the aggregation of the encoded protein. Resulting plaques accumulate within the glia and motor neurons, damaging them through an as-of-yet not fully understood mechanism.^87,88^ Tofersen degrades the *SOD1* mRNA and reduces the amount of superoxide dismutase 1 in the cerebrospinal fluid.^89,90^ Currently ongoing clinical trials are investigating whether this reduction is sufficient to slow the progression of ALS.

The targets of these four clinically approved RNase H-inducing oligonucleotides have much in common, which indicates preferential targets as well as the potential limitations of these therapeutic modalities. All of them target proteins with toxic gain-of-function mutations.

Mipomersen, inotersen, and volanesorsen target proteins that are involved in lipid transport and/or metabolism, synthesized predominantly or exclusively in the liver.^91−94^ A number of RNase H-inducing oligonucleotides currently in clinical trials fall into the same group, with degraders of triglyceride level modulator *DGAT2* and lipid carrier lipoprotein(a) approaching or currently in phase III clinical trials. This highlights that delivery to organs besides the liver remain a major hurdle.^95^ Nucleic acid delivery vehicles have the potential to overcome this, but even so, currently available strategies are limited.^96^ Altogether, RNase H-inducing oligonucleotides are one of the prime strategies to go after targets produced in the liver as well as targets difficult to modulate on a protein level, like extracellular proteins with poorly defined binding surfaces.^97^

### 2.2 RNA Interference and siRNA

RNA interference (RNAi) is another oligonucleotide-guided pathway through which RNA can be targeted and inactivated or degraded.^98,99^ It can utilize two distinct types of guide RNAs with distinct biogenesis and mechanisms of action–micro RNAs (miRNAs) and small interfering RNAs (siRNAs).^100^ The former are endogenously found in most eukaryotes, where they are produced as 70–100-nucleotide-long pri-miRNAs, which get processed into pre-miRNAs and finally active miRNAs, RNA duplexes of 19–25 nucleotides. siRNAs, on the other hand, are present in plants and various lowereukaryotes. They have several biogenesis pathways and are found as RNA duplexes of 21–23 nucleotides, although curiously they can be utilized by the human RNAi system, which is the enabling factor for siRNA therapeutics.^101,102^ miRNAs act primarily as translation repressors, and a single miRNA has many targets, whereas siRNAs are a part of response against pathogens and transposons and have only a single target. miRNA sequences are only partially complementary to their targets, and they do not typically induce degradation, whereas siRNA sequences perfectly match those of their targets, which are primed for degradation.^101,103^

Differences aside, their molecular mechanisms of action are similar as they both get loaded onto RNA-induced silencing complex (RISC).^104^ The formation of this complex is initiated when one of the argonaute proteins binds either miRNA or siRNA duplex, followed by ejection of the passenger strand. The remaining guide strand remains bound to an argonaute protein, which then forms the core of the RISC complex; this mature complex will then bind its target RNA, as defined by the guide. There are several mechanisms through which the target gets transcriptionally repressed or degraded; importantly, some argonaute proteins like human AGO2 act as endonucleases, leading to direct cleavage of RNA.^105^ Interestingly, the domain with endonuclease activity in AGO2 is closely related to that of RNase H—it contains three acidic (one aspartate and two glutamate) residues which bind two divalent cations such as magnesium that activate water, resulting in cleavage of the correctly positioned RNA, although in this case, it has to be a guide RNA:target RNA duplex.^106^ Similarly to RNase H, AGO2 has strict structural requirements for the cleavage; hence, modifications on the loaded siRNA must not alter the geometry of the RNA:RNA duplex.

**Figure 4:**
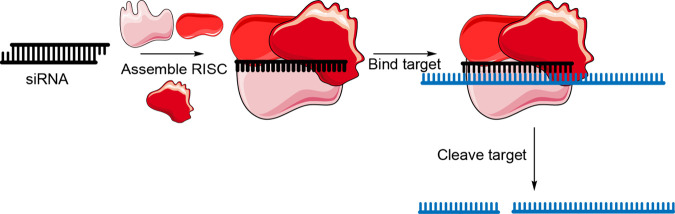
Mechanism of siRNA-induced RNA degradation.

The tunable mechanism of siRNA systems has led researchers to adapt these tools for both basic research and therapeutic applications. In the most used iteration of this system, cells are transfected with an siRNA duplex, the guide (antisense) strand gets loaded onto AGO2 in the RISC complex, whereas the passenger (sense) strand gets ejected (Figure 4). Then the siRNA-loaded RISC complex will bind and cleave RNAs complementary to the guide strand, thus reducing the levels of target RNA and, if it is a coding RNA, the corresponding protein.^17^ Both siRNA strands are composed of modified RNA; thus, the RISC complex must be able to differentiate the guide and the passenger, and this is achieved by engineering asymmetric strands with differential overhangs and thermodynamic stability.^107−110^

The modification landscape of siRNAs is similar to that of RNase H-inducing oligonucleotides, with some key differences. Most impactfully, only a limited number of phosphorothioate linkages can be tolerated on siRNAs; thus, they are typically engineered only on the flanks of both the guide and the passenger strands.^111^ As these linkages are key to serum protein recruitment, siRNAs will interact with them much less than with RNase H-inducing oligonucleotides and thus see much faster clearance rates and poorer trafficking as well as cell entry.^112−114^ As such, siRNAs require more elaborate delivery strategies, which will be discussed below. Furthermore, they require additional protection from ribonucleases, which is achieved by exchanging the 2′-OH group on ribose backbones into 2′-Fluoro or 2′-OMe.^17^ Many ribonucleases cleave RNA through deprotonation of the 2′-OH group; thus, transforming it into a different group confers complete protection against this mechanism of degradation.^33^ Both 2′-Fluoro and 2′-OMe protect siRNA from serum endonucleases, are similar enough in size to 2′-OH to retain activity in the RISC complex, whereas 2′-MOE, favored in RNase H-inducing oligonucleotide, is likely too bulky for this application.^115−117^ The 2′-Fluoro group forces the ribose to the C3′-endo position and greatly improves its binding activity toward its partner, which can increase the potency of the guide RNA.^118^ However, too many 2′-Fluoro modifications on a siRNA duplex will increase its stability so it will become impossible to dissociate it, thus ablating its activity. As such, in state-of-the-art siRNAs, a mixture of 2′-Fluoro and 2′-OMe is used to obtain optimal binding activity—strong enough for active RISC complex to find its target RNA but not too strong to prevent dissociation and loading of siRNA duplex.

As mentioned above, siRNA duplexes exhibit limited interactions with plasma proteins and hence are rapidly cleared.^112^ As such, they require more elaborate delivery strategies. To date, two strategies in siRNA delivery have seen success in the clinic—lipid nanoparticles (LNPs) as delivery vehicles and GalNAc ligands for selective internalization into hepatocytes.^119,120^ LNPs, as the name suggests, form lipid envelopes around the siRNA to be delivered, which can merge with cells and, once inside, promote escape from endosomes and thus release the siRNA into cytosol of the cell. Typically, LNPs consist of several different lipids which carry out a number of functions—positively charged lipids which interact with the negatively charged RNA, ionizable lipids which become positively charged and promote escape from acidic endosomes (pH 5.0–6.5), PEGylated lipids which improve circulation properties and prevent capture by phagocytes, as well as phospholipids and cholesterol to stabilize LNP structures.^121^ As with many other delivery approaches, most of the payload ends up being delivered to the liver, with ability to target different tissues being an area of intensive research.^122^ GalNAc functions in a very different way—it is a sugar ligand for a receptor found on hepatocytes; interactions with this receptor result in internalization into cells.^123^ This is a core principle behind GalNAc–siRNA conjugates, which typically contain a trimer or a tetramer of GalNAc ligands conjugated to the passenger strand of an siRNA, which then gets localized to hepatocytes expressing the asialoglycoprotein receptor.^124^ This allows for effective mRNA degradation in hepatocytes. However, it remains to be seen whether different cell types and tissues can be targeted by using ligands for different receptors.^125^

siRNAs have found success in the clinic, with six approved both by the FDA and EMA to date (Table 3).^126−131^ All six target mRNAs were made in liver cells, with Patisiran being delivered as siRNA formulated in LNPs and the remaining five as GalNAc conjugates. The patisiran siRNA is has 21 nucleotides in both the guide and passenger strands, with some of them modified with the 2′-OMe group—as LNPs provide protection from serum nucleases, siRNAs packaged this way need not be heavily modified to confer nuclease protection.^132^ Nedosiran has 22 nucleotides on the guide and 36 on the passenger strand, with the other four having 23 and 21 nucleotides, respectively, with the passenger strand conjugated to the GalNAc trimer or tetramer. In the case of Nedosiran, the four GalNAc units are conjugated to the 2′-O groups on four nonbase pairing adenosines through an amide linkage, while in the other four, a GalNAc trimer is attached to a phosphate on the 3′ end. They carry many more modifications than the LNP-formulated patisiran as they do require serum nuclease protection, having between one and four phosphorothioate linkages on the flanks of both guide and passenger strands, and all the 2′-OH groups changed into 2′-OMe or 2′-F, with high modification densities having been shown to dramatically increase serum stability.^115^

**Table 3:** Approved siRNA Therapeutics

Name of the siRNA	First approval year	mRNA target	Indication	Delivery strategy	Administration
Patisiran (Onpattro)	2018	*TTR*	Hereditary transthyretin mediated amyloidosis	Lipid nanoparticles	Intravenous, every 3 weeks
Givosiran (Givlaari)	2019	*ALAS1*	Acute hepatic porphyrias	GalNAc	Subcutaneous, monthly
Lumasiran (Oxlumo)	2020	*HAO1*	Primary hyperoxaluria type 1	GalNAc	Subcutaneous, every 1 or 3 months
Inclisiran (Leqvio)	2020	*PCSK9*	Hypercholesterolemia	GalNAc	Subcutaneous, every 6 months
Vutrisiran (Amvuttra)	2022	*TTR*	Hereditary transthyretin mediated amyloidosis	GalNAc	Subcutaneous, every 3 months
Nedosiran (Rivfloza)	2023	*LDHA*	Primary hyperoxaluria type 1	GalNAc	Subcutaneous, monthly

Patisiran, LNP-formulated siRNA and vutrisiran, a GalNAc conjugate, have both have been approved against the same disease and have the same target—hereditary transthyretin mediated amyloidosis and degrading the mRNA of transthyretin, just as RNase H-inducing Inotersen.^133,134^ The mechanism of action of transthyretin mRNA degradation is discussed in Section 2.1. While all three afford a similar reduction in circulating transthyretin levels, they have different administration regimens—inotersen is administered once every week subcutaneously, patisiran is administered once every 3 weeks and, owing to LNP formulation, intravenously, whereas vutrisiran is administered once every three months subcutaneously.^135^ Due to siRNAs having long half-lives once loaded onto RISC, they remain active for longer compared to RNase H-inducing oligonucleotides.^136^

Givosiran is GalNAc conjugate that has been approved against acute hepatic porphyrias and targets *ALAS1*, encoding delta-aminolevulinic acid synthase, the enzyme which catalyzes the first and rate-limiting step of heme biosynthesis.^137^ The genetic cause for acute hepatic porphyrias is deficiency for enzymes downstream of delta-aminolevulinic acid synthase, which can cause acute episodes where overexpression of hepatic *ALAS1* is induced, but the downstream biosynthetic defects lead to accumulation of intermediate products and overall depletion of heme. This leads to a negative feedback loop, causing the further overexpression of *ALAS1*. This results in a build-up of neurotoxic intermediates such as 5-aminolevulinic acid, the driver of this disease.^138^ Givosiran relieves this buildup by closing this negative feedback loop via reduction of delta-aminolevulinic acid synthase and its toxic product 5-aminolevulinic acid levels.^139^

GalNAc-siRNA conjugate lumasiran has been approved against primary hyperoxaluria type 1, and targets and degrades mRNA encoding hydroxyacid oxidase 1. The key hallmark of this disease is increased excretion of oxalic acid, which leads to buildup of insoluble calcium oxalate and formation of kidney stones.^140^ In healthy cells, glycolic acid is transformed into glyoxylate by hydroxyacid oxidase 1, which is further metabolized into glycine by alanine:glyoxylate aminotransferase or into oxalate by hepatic lactate dehydrogenase. People affected by primary hyperoxaluria type 1 have genetically debilitated alanine:glyoxylate aminotransferase; thus, the metabolic intermediate glyoxylate can only be transformed into oxalate, which is the cause of its buildup. Lumasiran reduces the levels of the enzyme that catalyzes the glyoxylate-to-oxalate transformation, and leads to normalization of urinary and plasma levels of oxalate.^141^ Nedosiran also targets the same pathway and has been approved against the same indication as well as to lower urinary oxalate levels in children but degrades a different target—hepatic lactate dehydrogenase, encoded by the gene *LDHA*.^142^ As nedosiran targets the enzyme that catalyzes the final step of oxalate synthesis, it potentially has a wider scope of applicability and can be effective against gain of function mutations in both *LDHA* and upstream genes. Like lumasiran, it leads to normalization of urinary and plasma oxalate levels.^142,143^

Inclisiran, also a GalNAc conjugate, is an approved therapy against hypercholesterolemia and functions by degrading proprotein convertase 9, encoded by gene *PCSK9*. This secreted protein is responsible for maintaining the levels of low-density lipoprotein (LDL) receptors on hepatocytes by catalyzing their internalization and degradation. These receptors facilitate clearance of cholesterol-carrying LDL particles from the blood.^144^ Gain of function mutations in the *PCSK9* make it more efficient in degrading the LDL receptor, which in turn makes clearance of LDL particles from blood less efficient and increases the levels of circulating cholesterol, the underlying reason behind hypercholesterolemia.^145^ Notably, people with a loss of function mutation in the PCSK9 gene were found to have the negative phenotype, with lower circulating LDL levels and resistance to associated cardiovascular diseases.^146^ Inclirisan functions in a similar manner—it lowers the levels of *PCSK9* mRNA levels in hepatocytes, which reverses the elevation of LDL levels.^147,148^

The approved siRNA therapeutic landscape has much in common with RNase H-inducing oligonucleotides, which is not surprising given the overall similarity in these modalities.^149^ siRNA therapeutics do have a unique advantage—as the siRNA:RISC complex is stable for days or weeks, siRNA does not need to be administered as frequently as most other therapeutic modalities, e.g., inclirisan is administered twice a year.^148^ Having good success with treating pathologies associated with gain of function mutations in the liver, it remains to be established whether this can be recapitulated in other tissues—perhaps the biggest limitation of this approach. Toxicity associated with off-target degradation also remains a concern and is one of the reasons why these modalities fail in clinical trials.^125,150,151^ Progress is being made in solving both problems with improvements in delivery vehicles, chemical modification landscape, and sequence optimization. With a number of siRNAs targeting different tissues being in late-stage clinical trials, it is likely that first nonliver-targeting siRNAs will see approval in the near future.^143,152−154^

Delivery of siRNAs into cells is not the only way to achieve RNAi-induced targeted RNA degradation—a DNA plasmid that encodes instructions to make siRNA precursors can be delivered instead. This is typically achieved by encoding a small hairpin RNA (shRNA), which is exported to the cytoplasm, processed into siRNA, and loaded onto the RISC complex by Dicer ribonuclease.^155^ Plasmids encoding shRNA are one of the most common technologies to establish conditional gene knockdown in tissue culture.^156^ This approach has not yet seen success in the clinic, at least in part due to a need of suitable delivery vectors and safety concerns about delivery of DNA plasmids which can integrate into the host genome.^157^ Nonetheless, therapeutic potential has been demonstrated in mice; thus, shRNA vectors do have the potential to join the ranks of therapeutically useful RNA degradation agents.^158^

### 2.3 CRISPR/Cas-Induced RNA Degradation

Ever since the first demonstrations of successful gene editing using Cas9, CRISPR has become a ubiquitous tool for nucleic acid manipulation.^159,160^ In nature, clustered regularly interspaced short palindromic repeat (CRISPR)-Cas systems are utilized by bacteria and archaea as acquired immunity systems against undesirable nucleic-acid-based materials, including phages, transposons, and plasmids.^161^ The core component of this system is the CRISPR array—a collection of nucleic acid sequences for acquired immunity. In cells possessing this system, certain pathogenic nucleic acids will get cut and integrated into the CRISPR array, which is carried out by Cas family proteins such as Cas1 and Cas2.^162^ These sequences then get expressed and processed into mature guide RNAs for Cas family nucleases, such as Cas9 or Cas13.^18^ The next time the same undesirable nucleic acid agents enter cells, the Cas nucleases will degrade them.

A number of Cas nucleases have been reported to degrade RNA, with Cas13 (Cas13a formerly known as C2c2) being perhaps the most well-characterized one.^150^ The RNA-cleaving site of Cas13 contains two HEPN domains, with each one having one histidine and one arginine residue in the active site.^163−165^ As such, Cas13 has an RNA degradation mechanism distinct from RNase H. Rather, it is akin to RNase A, explored more in-depth in Section 2.6.^33^ Interestingly, the binding site for guide RNA (crRNA) is not adjacent to the cleavage site. As a result, the bound RNA retains some flexibility and thus the cleavage can occur on several locations on the same transcript.^164^ Upon certain levels of infection, Cas13 will start exhibiting collateral cleavage, e.g., will start to degrade endogenous RNA in a nontargeted manner, which will put the infected cell into a dormant state.^166^ The nonselective cleavage is only triggered in the presence of the targeted sequence, e.g., in the presence of a pathogen. This mechanism prevents phages from replicating continuously and acquiring Cas13 immunity, thus allowing the host organisms to protect their populations from immune-system-evading pathogens.

Cas13 is a part of the class 2 CRISPR/Cas system, meaning that Cas13 is the only nucleic acid effector. A number of multieffector class 1 systems can also induce RNA cleavage.^167,168^ One of the best characterized class 1 ribonucleases, Csm6, is typically found in CRISPR systems which degrade both pathogenic DNA and RNA.^169^ Akin to Cas13, it possesses an HEPN domain with histidine as the key residue for RNA cleavage. As two HEPN domains are required for RNA cleavage, Csm6 is active only as a dimer.^170^ It is also a collateral activity ribonuclease, and this activity is controlled by other proteins in the class 1 system, which produce signaling molecules which affect the overall conformation of Csm6 and the trans-cleavage ability.^171,172^ Csm3, another protein in the same family, is also a ribonuclease.^168^ It does not contain the HEPN domain or histidine residues in its active site. Instead, it has a number of highly conserved aspartate and/or glutamate residues and its RNase activity is divalent metal ion-dependent, thus it can be speculated to use RNase H-like mechanism.^173^

Class 2 Cas proteins, in addition to Cas13, have also been shown to exhibit RNase activity. Cas9 came to be associated primarily with gene editing, although it can also act as an RNA-guided ribonuclease when provided with suitable guide RNAs.^174^ The cleavage site in Cas9 contains glutamate, aspartate, and histidine residues which bind divalent metal ions, and thus Cas9 cleaves nucleic acids in a similar manner as RNase H.^175^ Cas9 is both a DNase and an RNase, which serves as an example that the water activation mechanism can be utilized to cleave either of the nucleic acids. This highlights that conformational specificity is key for nucleases that cleave only one type of a nucleic acid duplex, which is the case for RNase H but not Cas9. Some Cas12 family enzymes have also been demonstrated to act as RNA-guided ribonucleases.^176,177^ Akin to Cas9, they possess an RNase H-like active site.

CRISPR/Cas systems and, in particular, Cas13 have been adapted for various RNA cleavage applications. It should be stated that these systems have one key disadvantage over RNase H-inducing oligonucleotides and siRNA—as CRISPR/Cas systems are not native to eukaryotic cells; they require delivery not just of guide oligonucleotides but also the Cas proteins or plasmids that encode them. As such, delivery of these systems is more complicated compared to technologies reliant on oligonucleotide payloads.^178^ Furthermore, many of the Cas enzymes also exhibit collateral effect, which is undesirable for therapeutic development.^179^ Significant progress has been made in developing high fidelity Cas13 enzymes as well as systems with negative feedback loops for Cas13 expression, both of which minimize the off-target cleavage.^180,181^

Despite the limitations, Cas13 systems can be advantageous over other methods for specific applications. In one of the first studies showing applicability of Cas13, Abudayyeh et al. demonstrated that this enzyme can achieve knock-down of long noncoding RNAs *MALAT1* and *XIST* which are localized in the nucleus, whereas siRNAs against the same targets had minimal effect.^18^ It has also been demonstrated that the Cas13-based system can achieve a more complete as well as more selective knock-down of mRNAs compared to an RNAi method, surprising given the known off-target effects of Cas13.^182^ It must be noted that these comparisons are somewhat limited in their scope as efficiency of both systems can be modulated by altering the targeting sequence as well as modifications on nucleic acid payload.

The collateral activity of CRISPR-associated RNases can also be taken advantage of, especially in the context of RNA detection. The group of Feng Zhang have developed a nucleic acid detection strategy called Specific High sensitivity Enzymatic Reporter unLOCKing (SHERLOCK).^183,184^ It relies on an RNA reporter that generates a signal upon cleavage, e.g., RNase Alert which becomes fluorescent. In the presence of the target RNA, Cas13 will start cleaving all the RNA in the mixture due to the collateral effect, including the reporter. This assay was further refined to enable mobile, lateral flow-based detection of viral infection.^185^ Combining similar principles with a microfluidics workflow, Sabeti and colleagues have developed CARMEN-Cas13, which pools samples to allow simultaneous high-sensitivity detection of many viral RNAs.^186^ They demonstrated the ability to differentiate between different types of influenza in patient samples as well as HIV stains with different mutations in their reverse transcriptase.

Cas13-based diagnostic methods were clinically tested during the COVID-19 pandemic. The SHERLOCK method was combined with loop-mediated isothermal amplification (LAMP) and the resulting assay STOP involved immersion of a swab in an extraction buffer, buffer exchange using magnetic beads, a single 60 °C heating step, and use of detection strips or a fluorescence reading to provide a result. This method was cross-validated against 402 patient samples (202 positive, 200 negative) previously tested via RT-qPCR.^187^ For 385 samples, the two methods were in agreement. STOP was also demonstrated to be more sensitive, e.g., to require less viral load for a positive prediction.

In another study, Uttamapinant and colleagues developed a method where SARS-CoV-2 RNA is amplified using a reverse transcriptase and T7 RNA polymerase.^188^ The resulting RNA mixture is then analyzed via SHERLOCK, via either a fluorometric readout or a lateral flow test. The latter utilized a fluorescein-biotin construct with an RNA linker; in a SARS-CoV-2-positive sample, it would get cleaved, and the liberated fluorescein would migrate to an upper band, serving as a positive signal. Both iterations of this method were cross-validated against 154 patient swab samples (81 positive, 73 negative) previously tested via RT-qPCR. It was found that the lateral flow version of this test agrees with RT-qPCR for 93.5% of the patients, whereas for the fluorescent readout test, the agreement was 98.1%. Altogether, these studies demonstrate that Cas13-based degradation methods are clinically useful for viral pathogen detection.

**Figure 5:**
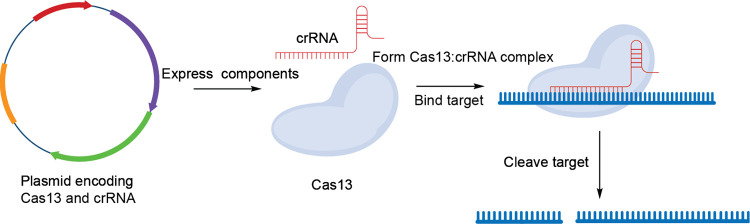
Mechanism of Cas13-based therapeutics. This class of therapeutics can be administered either as a plasmid or as a ribonucleoprotein complex made of Cas13 and crRNA.

Although CRISPR-Cas-based therapeutic RNA degraders have not made it to the clinic, several studies on animals have had promising results. Zhang et al. report tumor-specific apoptosis-inducing dual-locking nanoparticles, which selectively deliver Cas13 into tumor cells, and the resulting collateral cleavage induces widespread RNA degradation and tumor cell apoptosis.^189^ The key component of these particles is a plasmid that encodes Cas13 together with crRNA against the mRNA of PD-L1 (Figure 5). This plasmid is packed onto a positively charged polyethyleneimine (PEI) shell modified with various small molecules which prevent the uptake of these particles.^190^ In the presence of reactive oxygen species and a slightly acidic environment, characteristic to the tumor microenvironment, these small molecules get detached from PEI, which allows the nanoparticles to enter cells.^191^ After entering the tumor cells, the nanoparticles release the plasmid, and the Cas13-crRNA against PD-L1 mRNA system is expressed. After Cas13 finds its target, the collateral cleavage mechanism is triggered, and the resulting widespread RNA degradation triggers apoptosis. PD-L1 is a biomarker of a number of cancers and is not present in the majority of healthy cells; thus, this system should remain inert in nontumor cells, to prevent activity on healthy cells.^192^ The presence of PD-L1 overexpressing cells makes the tumor immuno-silent; thus, inactivation of these cells will enable immune cells to infiltrate the tumor and drive its regression.^193^ These DLNPs were tested in mice harboring PD-L1-positive tumors and were demonstrated to significantly slow down its growth. As further proof of specificity, these particles were tested in immunocompromised mice, as well as mice harboring a PD-L1-negative tumor. In both cases, no antitumor effect was observed.

Cas13 systems were also shown to have potential as antivirals. Blanchard et al. report inhalable nanoparticles which deliver Cas13 mRNA and crRNA against vital genes in influenza virus and SARS-CoV-2.^194^ They were packaged into nanoparticles using poly(beta amino esters) (PBAEs), a positively charged polymer demonstrated to be suitable for delivery of RNA payloads into lungs of mice.^195^ For delivery, these particles are nebulized and then inhaled. Upon entry into lung cells, they release their payloads into the cytosol, where Cas13 gets expressed. The active Cas13:crRNA complex then exerts an antiviral effect by degrading the targeted viral genes. They were tested against an influenza infection model in mice and were found to induce a 10-fold reduction of viral load in the lungs, as measured by qPCR and partially prevent weight loss caused by the infection. These nanoparticles with appropriate crRNA were also tested against a SARS-CoV-2 model of infection in Syrian hamsters. That led to a more modest, 2-fold reduction of the viral load in the lungs likely due to aggressiveness of the infection model. The fully treated group saw a weight progression similar to that of the uninfected group. A study by Abbott et al. has also demonstrated the utility of Cas13 as a prophylactic against SARS-CoV-2 as well as influenza A virus and MERS-CoV, although only in cellular models of infections and only in cells expressing Cas13d.^196^

Despite the demonstration of utility in animal models, it remains an open question whether using Cas13 or other CRISPR-associated RNases for targeted RNA degradation is advantageous over other oligonucleotide technologies. Perhaps the key disadvantage for these systems is the need to deliver both the crRNA and the Cas nuclease (or a plasmid or mRNA that encodes it). As discussed previously, Cas13 has been delivered both in mRNA and plasmid form when packaged with a positively charged polymer.^189,195^ Lipid nanoparticles are another type of vehicle that can be used for delivery of Cas13 in nucleic acid form.^197^ There is also a possibility to deliver an intact Cas13:crRNA complex, which has been successfully achieved via using a zwitterionic polymer.^198^ If these outstanding issues get successfully resolved, CRISPR-associated ribonucleases will have their chance to shine due to their ability to induce a nearly complete knockdown and efficiently degrade nuclear transcripts.

### 2.4 RNA Quality and Translational Control Mechanisms

Eukaryotic cells have evolved a number of RNA quality control mechanisms to ensure streamlined translation, both to conserve resources and avoid production of defected, potentially toxic proteins.^199,200^ These mechanisms can be taken advantage of to induce targeted RNA degradation. Some of these controls are related to the RNA structure and sequence—certain RNA species possess 5′ Cap structures and poly(A) tails, defects in which will lead to ribonuclease-induced degradation. mRNAs without or with defective caps get readily degraded by XRN family 5′-exonucleases.^201,202^ The poly(A) tail protects RNA from 3′ exonucleases through recruitment of poly(A)-binding proteins (PABPs), which block access to the 3′ end of RNA.^203,204^ Interestingly, binding of mRNAs by PABPs can be dependent on ongoing translation.^205^ Consequently, mRNA that stops translating protein will lose protection conferred by PABPs and get degraded.

Other controls are related to the process of translation and thus are relevant only to mRNAs, namely no-go decay (NGD), nonstop decay (NSD), and nonsense-mediated decay (NMD).^200^ The biological role of these mechanisms is to ensure that the protein synthesis is progressing normally and to prevent translation of prematurely terminated peptides.^206^ No-go decay is induced when translating ribosomes are stalled on the mRNA; this can happen due to translated mRNA possessing a secondary structure.^207^ Stalling results in collision of multiple ribosomes, which is followed by their ubiquitination and cleavage of the mRNA by a endoribonuclease.^208^ In yeast, this is done by Cue2, a homologue of mammalian N4BP2.^209^ The resulting RNA fragments were then degraded by XRN exonucleases and the exosome. The role of nonstop decay is to degrade RNAs which lack stop codons.^210^ In their absence, the ribosome will start translating the poly(A) tail, which results in poly lysine on the growing polypeptide, which stalls in the exit tunnel of the ribosome due to its positive charge.^211^ The stalled ribosome is recognized by dedicated proteins which, similarly to NGD, result in cleavage by endosomes, followed by degradation carried out by Xrn1 and exosomes.^212^

The role of nonsense-mediated decay is to degrade mRNAs with premature termination codons (PTCs).^213^ In some cases, PTCs have a direct link with disease—nonsense mutations in beta-globin (a cause of beta-thalassemia) result in a toxic version of this protein; thus, NMD protects from its build-up.^214^ In different contexts, NMD can be the underlying cause of the disease, as is the case for Duchenne muscular dystrophy, where a PTC is found in dystrophin gene.^215^ Although the truncated version of this protein is still functional, NMD results in degradation of its mRNA and depletion of the protein.^216^ This process can be targeted therapeutically—ataluren is a drug which suppresses NMD and, in the case of dystrophin, promotes translation of the truncated-yet-functional protein.^217^ NMD is a multistep process which begins with detection of the PTC. The full mechanism is not yet completely understood, but it is known to be initiated by a stalled exon junction complex.^218^ It leads to recruitment of the NMD regulator proteins and phosphorylation of the master regulator UPF1, an RNA helicase.^219,213^ This in turn results in recruitment of endonuclease SMG1, which carries out the initial cleavage of RNA.^220^ As is the case for most other RNA quality control mechanisms, the damaged mRNA is then degraded by Xrn1 and the exosome.^221^

The key players in the RNA quality control mechanisms are the 5′ and 3′ exonucleases, which degrade RNA molecules that did not pass the quality control checks. The main exoribonuclease that carries out 5′ to 3′ degradation is Xrn1, which possesses RNase H-like active site.^202,222^ The activity of Xrn1 differs from ribonucleases discussed previously as it is processive—it can completely chop up an RNA molecule rather than introduce a single cleavage site, and it does not release partially degraded transcripts.^223^ After each cleavage event carried out by Xrn1, the resulting mononucleoside phosphate is released from the active site and the resulting 5′-phosphate RNA is translocated, a process driven by electrostatic interaction between the 5′-phosphate and its binding pocket.^222^

Both structural and translational RNA quality control mechanisms have been utilized for RNA destabilization and targeted degradation and in many cases pioneered by Ionis Pharmaceuticals, although these approaches are yet to be tested in the clinic.^224^ Perhaps the first approach to be tested was decapping antisense oligonucleotides.^225^ An oligonucleotide targeting the 5′ end of ICAM-1 mRNA was reported; the oligonucleotide was conjugated to a europium complex, which was demonstrated to cleave triphosphates characteristic to RNA caps.^226^ The construct was shown to be able to decap mRNA in vitro, although they were only able to reach a quite modest efficiency of 26% with prolonged treatment. In a cellular model, this bifunctional was demonstrated to reduce the concentration of ICAM-1 protein by 90%, with the antisense oligonucleotide and the europium complex individually having a much milder effect, if any. This study has demonstrated that RNA quality control mechanisms can be utilized for modulation of RNA stability, although transfection of metal complexes poses many challenges that complicate translation to the clinic.

**Figure 6:**
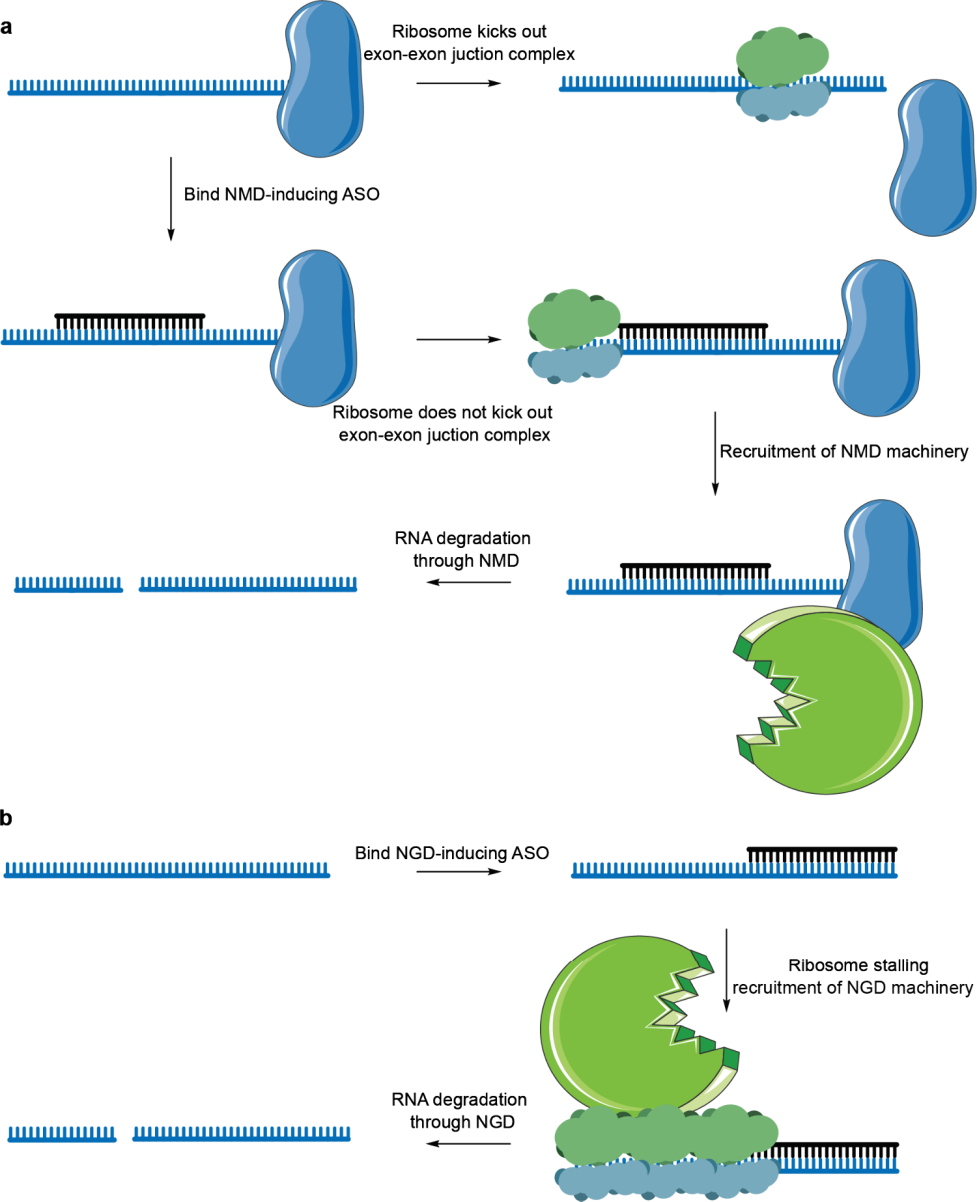
Oligonucleotides which induce targeted RNA degradation via RNA quality control mechanisms. (a) Targeted RNA degradation through induced nonsense-mediated decay (NMD). (b) Targeted RNA degradation induced through no-go decay (NGD).

Antisense oligonucleotides can also trigger RNA degradation via translational quality control mechanisms. A study by Ionis Pharmaceuticals demonstrated that nonsense-mediated decay-inducing oligonucleotides can be rationally designed.^227^ The reported oligonucleotides were fully modified with 2′-MOE groups, thus unable to induce RNase H or RISC. They were designed to modulate splicing; namely, to induce exon skipping and shift the reading frame so it would have a PTC which would trigger NMD (Figure 6a).^228^ Such oligonucleotides were successfully designed against mRNAs of *STAT3* and *Sod1*, and led to a decrease of both targeted mRNA and associated protein levels in cell culture and mouse models. Crucially, knocking down UPF1 or SMG6, two key factors in NMD, partially rescued the levels of the targeted transcripts, validating that this process is carried out by NMD machinery. Some prior studies have also reported hypermodified ASOs being able to decrease transcript levels potentially via NMD, although without a mechanistic validation.^229,230^

No-go decay has also been utilized by the team at Ionis Pharmaceuticals in design of RNA-degrading ASOs (Figure 6b).^231^ NGD-inducing oligonucleotides were designed in a similar manner as the NMD-mediating ASOs—they were hypermodified, thus unable to induce RNase H. A library of such oligonucleotides was screened against the mRNA of nucleolin, and both NMD- and NGD-inducing oligonucleotides were discovered. NGD-inducing oligonucleotides most likely stalled ribosomes by tightly binding the mRNA, with ribosomes being unable to displace it. The activity of these oligonucleotides was found to depend on PELO and HBS1L, two key factors in NGD, thus validating this mechanism of action.^232,233^

Altogether, these studies demonstrate that intricate RNA quality control mechanisms can be utilized to degrade RNAs with potentially therapeutic effect. However, it is not clear what advantages these approaches have over more established technologies of RNase H-inducing oligonucleotides and RNA interference. For translational quality control mechanisms, it is not known what proportion of mRNAs meets the sequence requirements, e.g., cryptic PTCs to be targeted. Furthermore, these mechanisms are only relevant to mRNAs, with noncoding RNAs not being subjected to the same quality controls. Much is left to discover both about noncoding RNAs and their quality control mechanisms; thus, designing oligonucleotides which utilize them might provide new opportunities to modulate their levels as well as functions.^234^

### 2.5 Direct RNA Degradation: Oligonucleotide-Based Artificial Ribonucleases

All the pathways described above are reliant on recruitment of ribonucleases to degrade RNA. However, that is not the only way—RNA can instead be brought into the proximity of an artificial construct resembling a ribonuclease. Very often, the guiding moiety in these species in an oligonucleotide, hence this group of degraders is called oligonucleotide-based artificial nucleases (OBANs).^235,236^ They typically mimic one of two groups of naturally occurring ribonucleases—transition-metal-dependent or general acid/base catalyst-dependent.^33^ The former group utilizes cations like zinc or manganese to activate water and hydrolyze the phosphodiester bond of RNA akin to the mechanism of ribonuclease H. One key difference is the metal binding matrix—carboxylate-side chain amino acids are sufficient for magnesium, whereas the binding matrix of transition metals employs softer electron pair donors, typically several histidine residues.^237^ The metal is another key difference—zinc and manganese are more potent nucleophilic activators of water compared to magnesium; thus, they reduce the p*K*_a_ of bound water more. Overall, this leads to faster kinetics of RNA cleavage.^238^

**Figure 7:**
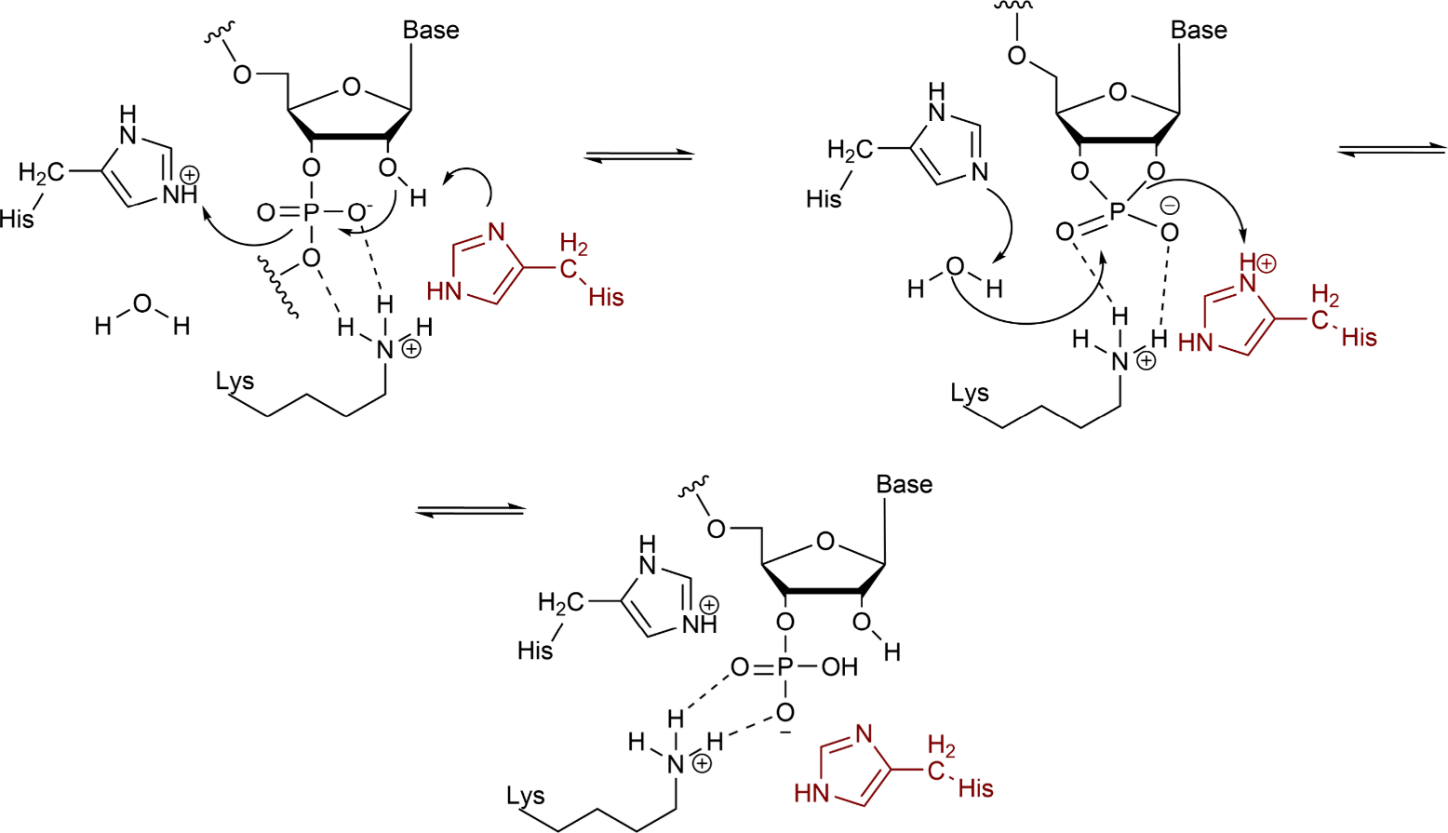
Simplified molecular mechanism of ribonuclease A.

As the name implies, general base/acid ribonucleases use basic and acidic residues to cleave RNA through deprotonation of the 2′-OH group. One of the most thoroughly characterized enzymes—ribonuclease A—belongs to this class.^239^ It utilizes a pair of histidines that act as bases to deprotonate 2′-OH group and water for initiation of hydrolysis, and acids to facilitate the leaving group bond cleavage (Figure 7).^240^ Another key residue in the RNase A mechanism is positively charged lysine, which coordinates the phosphodiester to be cleaved, counteracting its negative charge.^241^ In RNase H-type nucleases, magnesium is responsible for this role, but nucleases without metal cations in the active center require positively charged amino acids. This lysine was also demonstrated to form hydrogen bonds with the transition state of RNA to-be-cleaved.^242^

Histidine is the amino acid of choice for cytosolic general acid/base nucleases due to its p*K*_a_ value, being close to 6 for the two catalytic residues in RNase A.^243^ As this is close to the pH value of the cytosol, histidine is found in both deprotonated and protonated forms, meaning it can function as both a base and an acid catalyst. In more acidic environments, these histidines are fully protonated, whereas in basic ones they are fully deprotonated and unable to act as bases/acids, respectively, which explains why RNase A has a bell-shaped activity curve, with peak activity at pH 6.^244^ Curiously, in a solution, RNA has peak stability at pH 5, when the sum of acid-catalyzed and base-catalyzed hydrolysis rates reaches a minimum.^245^

**Figure 8:**
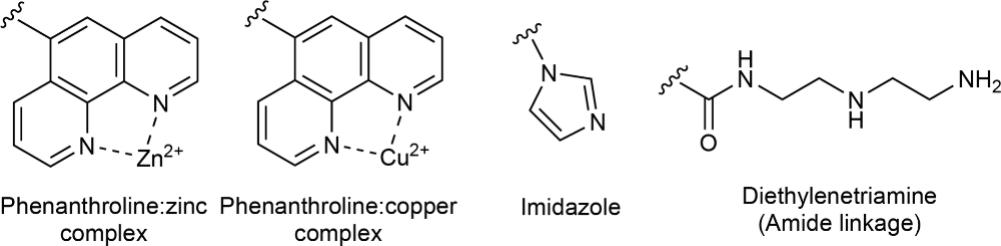
Examples of warheads utilized in the design of antisense oligonucleotide-based artificial nucleases.

Transition metal and general base catalysis are the guiding principles behind the design of most oligonucleotide-based artificial ribonucleases (Figure 8). The first oligonucleotide-based artificial RNase was reported by Chen and Sigman, which followed their previous work on artificial small molecule DNases, explored more in-depth in Section 3.3.^246,247^ These designs utilized the phenanthroline–copper(II) complex as the degrader warhead, joined via an amide linker to a guide DNA oligonucleotide. Although copper can activate water as a nucleophile, in this case a different mechanism was utilized—redox cycling. In the presence of a redox agent such as hydrogen peroxide or ascorbate, copper will cycle between oxidation states +2 and +1, generating radicals capable of RNA cleavage.^248,249^ This initial OBAN was designed to bind the 5′ end of *lac* mRNA and was demonstrated in vitro to degrade 20% of its target in 2 h. Crucially, no effect was observed when the phenanthroline warhead and the guide oligonucleotide were added separately, e.g., without covalent linkage, which shows that at least under the tested conditions, this phenanthroline-mediated cleavage is proximity-induced. The phenanthroline–copper complex was demonstrated being able to degrade oligonucleotides on its own; thus, its constructs should be used at low concentrations to avoid nontargeted RNA cleavage.^250^

Imidazole can catalyze RNA degradation both via binding transition metals as well as through acting as a general base/acid, with this theme explored in the first paper reporting imidazole-oligonucleotide conjugates. In this study, a single imidazole was attached to the 3′ end of an antisense oligonucleotide, and was tested as a degrader against a complementary sequence with overhangs on both 5′ and 3′ ends.^251^ The degradation reactions were carried out in vitro at pH 7.0 at room temperature. Interestingly, no metal-independent degradation was observed. However, adding 50 μM of zinc to this mixture was sufficient to induce the cleavage, with appreciable degradation observed in timeframes as short as one hour. The cleavage was observed at two sites, likely a consequence of a short two-carbon linker.

RNA degradation through pure general acid/base catalysis has also found application in the design of OBANs. One of the first reported degraders of this kind utilized a mechanism similar to that of ribonuclease A, using a pair of imidazoles or an imidazole–amine catalytic dyad, attached to a guide sequence through replacement of one of its nucleotides.^252,239^ As a result, the nucleotide in front of the degrader moiety in its binding partner was unpaired. The OBAN was found to be able to cleave the target strand in vitro—under mild conditions (25 °C, pH 7.2), 10% of the oligonucleotide was cleaved in 5 days. Interestingly, all of the cleavage was found to take place on the phosphodiester bond in front of the degrader moiety; it was postulated that is due to the vicinal base being unpaired and in a nonhelical form, although the limited reach of the degrader might have had an impact as well.^253^ Degrader constructs containing only polyamines have also been reported and were found to cleave target RNA faster under more basic conditions, closer to the p*K*_a_ values of the amine groups, which further supports the validity of the general base/acid mechanism.^254^

OBANs with phosphodiester backbones may be capable of undesirable autocleavage. Indeed, our own work has demonstrated that covalently attaching an imidazole warhead to an RNA oligonucleotide can induce its degradation.^255^ The most popular alternative to phosphodiester that circumvents this issue is the peptide bond, forming the core of peptide nucleic acids (PNAs).^256^ The first reported PNA-based OBAN utilized a diethylenetriamine as the warhead, with the amine groups acting as the general acid and base catalysts.^257^ In this case, the warhead was attached to the 5′ end of the guide oligonucleotide, and the degradation was localized in unpaired 3′ overhang of the target strand, opposite to the degrader moiety. In vitro reactions were carried out in pH 7 media at 40 °C, and the target RNA got degraded with a half-life of six hours. Constructs that utilize transition-metal-induced water activation were also explored, with the first report using a phenanthroline–zinc complex as the warhead.^258^ These constructs were found to cleave its target (a fragment of telomerase mRNA) site-specifically in vitro, with up to 30% degradation in 24 h. This study also found that a more flexible linker between the degrader and guide oligonucleotide is beneficial for degradation. More recent PNA-based OBANs that utilize phenanthroline–zinc warhead were reported to cleave target RNA more rapidly, with 7–8-h half-lives.^259^ This was achieved by engineering an unpaired bulge in the target RNA which is opposite the phenanthroline warhead. This study also found that adenosine-rich bulges get cleaved faster.

Oligonucleotide-based artificial ribonucleases have not yet made inroads into the clinic and, in fact, have been almost exclusively used in vitro, which can be attributed to several factors. The rates of RNA degradation reported so far are relatively slow, thus outcompeting natural RNA turnover might be an issue.^260,2^ For transition-metal-dependent OBANs, cells might not have sufficient concentrations of relevant cofactors to warrant degrader activity.^261^ OBANs with nonmodified backbones may not be sufficiently stable, whereas oligonucleotides with nonphosphodiester backbones may bind their target RNA too tightly and so be unable to deattach from the cleaved RNA thus not catalytic.^262^ As such, whether or not OBANs can outperform nuclease-recruiting oligonucleotides under some circumstances. Perhaps their biggest advantage lies in the fact that they do not rely on endogenous enzymatic machinery and thus are cellular context-independent, but given their limited success in cell culture experiments, it is not clear whether it can find use. Other therapeutic oligonucleotides have found success only after suitable RNA modification patterns were discovered—perhaps a more thorough exploration of the appropriate chemical space will have a similar effect on the composition of the OBANs.

### 2.6 Ribozymes

Ribozymes—enzymes composed of RNA—are another class of molecules that can induce targeted RNA degradation.^263^ Their RNA cleavage mechanism does not differ much from that of nucleases—ribozymes utilize a combination of divalent metal ions and nucleic bases which act as general base and acid catalysts that are used to cleave the phosphodiester bond, although ribozymes are orders of magnitude slower than their protein-based cousins.^264^ The p*K*_a_ values of nucleic bases vary a lot depending on their environment and in some cases get close to the physiological pH, which enables their activity as general base and acid catalysts.^265^ Out of several known types of RNA cleaving ribozymes, the hammerhead ribozyme was the most thoroughly explored as a potential therapeutic agent. The native ribozyme is 50–150 nucleotides long and is composed of three helices that are required for (auto)cleavage.^266^ The canonical cleavage site is on the molecule of ribozyme, on an unpaired nucleotide of the helical junction, so it cleaves itself. However, hammerhead ribozymes that can act in a trans fashion, e.g., cleave other RNAs, are also known, or in some cases these ribozymes can cleave a phosphodiester bond on the same RNA molecule but situated hundreds of nucleotides away.^267,268^ Ribozymes are found all across the tree of life—initially identified in plant viruses, they have been discovered all across the tree of life including mammals.^269,266,268^

Several topologies of hammerhead enzymes exist, but they all have the three helices, and the cleavage always takes place on a nucleotide on one of the helical stems.^270^ In addition to that, hammerhead ribozymes require magnesium for both correct folding as well as cleavage.^271,272^ Intriguingly, the observed magnesium concentration required for in vitro cleavage was often significantly higher than that found in the cells. It was demonstrated that this phenomenon can be at least partially explained by the dielectric constants of the solution.^273^ Typical buffers have higher dielectric constants than cellular media; Sugimoto and colleagues demonstrated that doping the reaction buffer with 20% organic solvents such as ethanol or ethylene glycol both lowers the dielectric constant and dramatically increases the cleavage rate of the ribozyme under low magnesium conditions. Szostak and colleagues have shown that reduction of magnesium requirements in vitro can also be achieved by crowding agents such as polyethylene glycol, although this was demonstrated for ribozyme-catalyzed RNA assembly rather than cleavage.^274^

**Figure 9:**
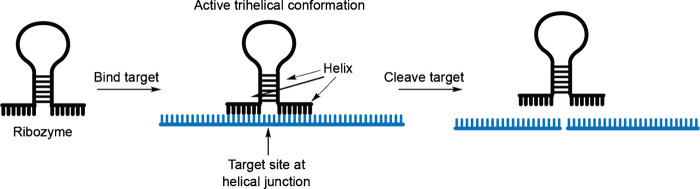
Mechanism of therapeutic trans-acting ribozymes.

Being nucleic-acid-based, ribozymes turned out to be programmable and were utilized for therapeutic applications. The first cases of artificial trans-RNA cleaving ribozymes essentially replicated the structure of the hammerhead enzyme on two RNA strands, which upon hybridization form the triple helical structure required for cleavage (Figure 9).^275,276^ Constructs in both studies were found to cleave RNA in the same position as natural hammerhead ribozymes on the stem of one of the loops, and their activity was found to be magnesium-dependent. Similar principles were utilized in the design of therapeutic ribozymes. Angiozyme is an angiogenesis inhibitor developed as an anticancer drug by Ribozyme Therapeuticals.^277^ This oligonucleotide drug was designed so it would hybridize with Flt-1 mRNA, resulting in the formation of the characteristic hammerhead ribozyme structure and cleavage of the mRNA strand. Angiozyme is heavily modified like most therapeutic RNAs, with most riboses harboring 2′-OMe groups and a number of phosphodiester linkages changed into phosphorothioates, and curiously, one of the cytosines harboring a 2′-C-allyl nucleotide. Angiozyme is formulated without a delivery vector, and thus it relies on local administration; its modification pattern was designed to protect it against serum nucleases.^278^ This ribozyme was demonstrated to cleave its target and prevent metastasis in a mouse model of colorectal carcinoma.^279^ This ribozyme construct entered clinical trials for solid tumors and metastatic breast cancer.^280,281^

Several other anticancer and antiviral ribozymes were designed. The first ribozyme to inhibit tumor growth in vivo was reported by Sioud and Sorensen.^282^ This ribozyme was designed to cleave the mRNA of PKCα, a protein kinase which promotes progression of various cancers.^283^ It followed similar principles to angiozyme—this ribozyme could cleave target RNA through formation of a trihelical junction. The construct was demonstrated to cut its target in vitro and prevent the progression of glioma in rat models. The successful application of this ribozyme against in vivo models is in large part owed to it being modified with 2′-amino groups, which protect the construct against nucleases but do not abolish its ability to cleave the target RNA. An alternative strategy to achieve targeted RNA degradation in vivo is to use delivery vectors. Fei et al. reported series of hammerhead ribozymes designed against survivin mRNA, packaged in adenoviral particles.^284^ Survivin is an antiapoptotic protein reported to promote angiogenesis, metastasis, and treatment resistance, thus its knockdown may suppress tumor growth.^285^ Antisurvivin ribozymes were demonstrated to cut RNA in vitro and in cell culture and slow down tumor growth in mouse models of hepatocellular carcinoma. The adenoviral vectors used to package the ribozymes provided both protection against ribonucleases and facilitated delivery into cells.

An alternative approach is to deliver a ribozyme-expressing plasmid, which has been used as a design strategy for antiviral ribozymes.^286^ In one case, hairpin ribozymes were designed against an overlapping region of *env* and *rev* genes in HIV. They were encoded in a plasmid and packaged in a lentiviral vector. These plasmids were transduced to various lymphoblast cell lines, and their resistance against HIV infection was tested. As expected, lymphoblast cells expressing the ribozyme were more resistant to infection than cells not expressing the ribozyme or expressing an inactive version. A modified version of this approach was tested in a clinical trial.^287^ Briefly, monocytes of AIDS patients were extracted, and CD4^+^-enriched T cells were purified and transduced with an anti-HIV ribozyme plasmid to enhance their survivability, followed by infusion back into patients. The procedure was well tolerated, and ribozyme-containing cells exhibited enhanced survival; however, the overall low ribozyme-containing cell count precluded further positive effects.^288^

The ribozyme platform bears many similarities to that of siRNAs—in both cases, the therapeutic agents are composed of modified RNA bases targeting a particular sequence. The two approaches have considerable differences too—siRNA therapeutics utilize endogenous cellular machinery to knock down RNAs, and this process overall is more efficient than the direct degradation induced by ribozymes, in part due to RNA cleavage rates being faster for protein-based ribonucleases.^289^ Furthermore, approved siRNA therapeutics retain their activity for weeks after administration, as RISC-bound RNA substrates are known to be exceptionally stable.^136^ Ribozymes, on the other hand, have much shorter half-lives, and thus, ribozyme-based therapeutics would need to be administered more often. Given this comparison, in most cases, the siRNA platform can provide superior solutions.

## 3 Small Molecule RNA Targeting

In addition to the establishment of siRNAs and RNase H-inducing oligonucleotides as therapeutic modalities, recent years have overseen a renaissance in targeting of nucleic acids with small molecules. Small molecules can circumvent numerous issues associated with nucleic acid therapeutics, as was discussed in the Introduction.^290^ The nature of targeting between the two classes is different—ASOs target a sequence, whereas small molecules target structures.^291^ The ability of small molecules to bind RNA selectively has been getting a lot of attention—recent studies coming both from academic institutions and biotechnology companies have demonstrated that high-throughput screening against RNA structures is indeed possible.^12,292−294^ It has also been demonstrated that numerous small molecules designed to bind proteins, including FDA-approved drugs, are also RNA binders.^295^ Methods for screening of RNA–small molecule interactions have also seen rapid advancement, with small molecule microarray screening as well as cross-linking and sequencing methods being some of the prime examples.^296−298^

Unsurprisingly, there is an ongoing effort to define both what is a good RNA target for small molecules and what small molecule features make them good RNA binders.^12,299−301^ As was explored in the previous parts of this review, binding is not always sufficient to exert a phenotypic effect, thus it is desirable to develop small molecules which can modulate stability of RNA. Indeed, small molecules can be envisaged to alter various properties of RNA, including localization, translational efficiency and epitranscriptomic modification.^302^ This part of the review will explore how small molecules were utilized to destabilize and degrade RNA, via recruitment of ribonucleases, direct RNA degradation, or structural destabilization.

### 3.1 Recruitment of Ribonucleases: RIBOTACs

The majority of approved antisense oligonucleotide therapeutics recruit ribonucleases, either RNase H1 or Ago2. Unsurprisingly, RNase recruitment approaches are being translated to the world of small molecules, with the most thoroughly explored ribonuclease in this context so far being RNase L (L stands for latent). In various eukaryotic cells, it serves as one of the pieces innate antiviral machinery.^303,304^ In noninfected cells, it is found in its inactive, monomeric form (latent). Infection of a cell with viral particles leads to a signaling cascade leading to its activation—type I interferon response is triggered by double-stranded viral RNA, which activates synthesis of rather unusual signaling molecules—2′-5′ oligoadenylates.^305^ RNase L binds these oligonucleotides, which triggers a conformational change in the protein, leading to the C-terminal ribonuclease domain getting exposed.^306^ This is followed by the dimerization of two ribonuclease domains, resulting in the active dimeric form of this enzyme. The key residues in RNase L are histidine, aspartate, and glutamate, and it is not transition-metal-dependent, thus likely follows RNase A-like general base/acid mechanism.^307^ In the early stages of RNase L activation, it will predominantly cleave viral and rRNAs, thus inhibiting expression of viral proteins.^308^ In the case of prolonged activation, the widespread rRNA damage will lead to apoptosis, the final line of defense against viral infection.^309^ Notice that this antiviral pathway holds many similarities with the bacterial Cas13, which relies on collateral RNA degradation to put the prokaryotic cells into stasis, thus preventing viral replication.^166^

**Figure 10:**
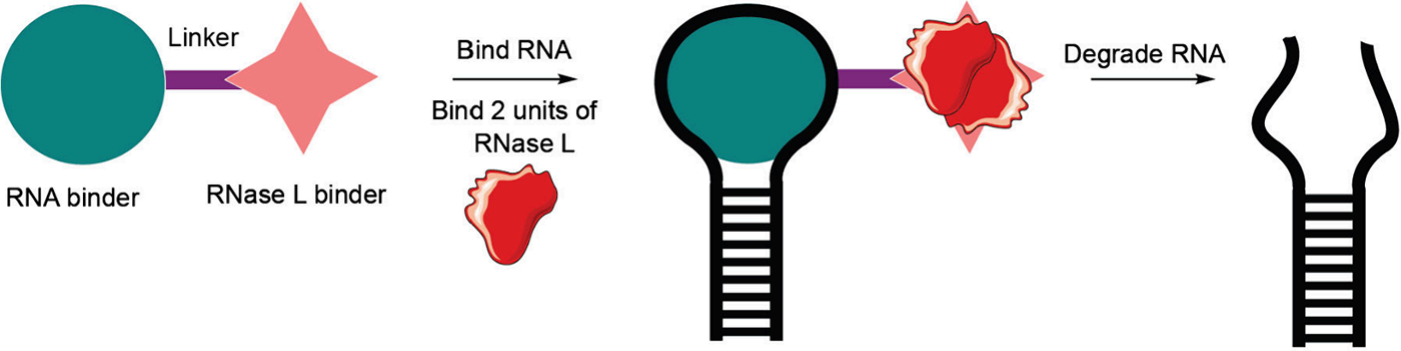
RIBOTACs degrade RNA by inducing proximity to RNase L.

RNase L has been utilized to design small molecule RNA degraders, ribonuclease targeting chimeras (RIBOTACs).^310^ These heterobifunctional molecules comprised of an RNA binder, a linker, and a moiety to recruit RNase L. The molecules function by bringing together a dimer of RNase L and the targeted RNA, resulting in its degradation (Figure 10). The construct in the first study reporting RIBOTACs targeted pri-miR-96 using a ligand previously developed by Disney and colleagues, connected to a 2′-5′ tetraadenylate through an amide- and PEG-based linker.^311^ This inaugural RIBOTAC was demonstrated to reduce the levels of pri-miR-96 by more than 50% across several cell lines in a ribonuclease L-dependent manner. Interestingly, there was little difference between RIBOTAC and its parent RNA binder molecule in affecting the levels of mature miR-96.

The initial construct had several limitations—the negative charge arising from the 2′-5′ oligoadenylate was compromising the molecule’s entry into cells, and their large molecular weight (2918 amu for the smallest RIBOTAC, roughly equivalent to 9-mer oligonucleotide) was not ideal for therapeutic applications. Given that these constructs were composed of a tetranucleotide conjoined to a relatively large RNA binder, they could be considered oligonucleotide–small molecule conjugates rather than small molecules. As such, their design was subsequently refined. Cruicially, it was discovered that the polyadenylate RNase L recruiter can be replaced by a small molecule without a pronounced negative charge.^312^ RNase L-recruiting small molecules were previously demonstrated to induce dimerization or RNase L as well as exert antiviral activity.^313^ Recently reported RIBOTACs also utilized smaller binder groups, which allowed the molecular weight of the construct to be reduced below 1,000 amu, in line with other heterobifunctional molecules such as PROTACs.^314^ It was also demonstrated that certain natural products can be transformed into RNA-degrading RIBOTACs.^294^ Zhang et al. reported series of G-quadruplex targeting RIBOTACs with a caged RNase L recruiter moiety, which could be released by a stimulus such as a biorthogonal reactant or elevated reactive oxygen.^315^ The utility of these inducible RNA degraders has been demonstrated in mice, where they acted as tumor growth suppressors, which shows that this modality has the potential for therapeutic application.

In an antiviral approach inspired by RIBOTACs, Min et al. demonstrated that RNase L-recruiting moieties can be appended directly to nucleotides.^316^ These constructs were demonstrated to exhibit anti-SARS-CoV-2 activity in cellular models of infections, as well as Syrian hamsters. They are postulated to function by getting incorporated into a growing viral RNA chain and then recruiting RNase L to degrade it. The study showed that sterically perturbing the structure of the RNase L recruiter leads to a significant but not a total drop in antiviral activity. This demonstrated that RNase L recruitment is the key mechanism of action, but other mechanisms, such as steric blocking, may also contribute. Various nucleotide derivatives are often phosphorylated exclusively by viral kinases thus are inactive in uninfected host cells; it remains to be seen whether that is the case with the RIBOTAC-like antiviral nucleotides.^317^

A number of RIBOTACs were demonstrated to work in cellular models, but examples of in vivo activity are limited. This could be due to intrinsic limits to their efficiency—in reports so far, they typically achieve close to 50% reduction of the targeted RNA, which might be not sufficient to exert a phenotypic effect. Their relatively large size is likely translating to a poor pharmacokinetic profile and limited tissue penetration. So far, all examples of RIBOTACs utilize ribonuclease L. It has been reported to exhibit an uneven tissue distribution, with high expression levels only in the spleen, lungs, and thymus, organs prone to viral infections.^318^ As such, molecules designed for different tissues would require the recruitment of natively expressed ribonucleases.

### 3.2 Small-Molecule-Induced RNA Degradation through Quality Control Mechanisms

As discussed in Section 2.4, eukaryotic cells have evolved numerous RNA quality control mechanisms that may be triggered through purposely designed antisense oligonucleotides. Small molecules have also been demonstrated to induce targeted RNA degradation through splicing modulation.^319^ The foremost example is branaplam, developed by Novartis.^320^ This molecule was initially reported as a splicing modulator of *SMN2*, a gene with a key role in spinal muscular atrophy. In the disease state, a genetic mutation causes exclusion of exon 7 from the mature *SMN2* mRNA, which in turn results in production of nonfunctional protein.^321,322^ Branaplam was found to reverse this, and force inclusion of the exon 7 by acting as a molecular glue between *SMN2* mRNA and U1 snRNP complex, a part of the spliceosome.^323^ In this case, the outcome is the production of a functional protein rather than degradation.

**Figure 11:**
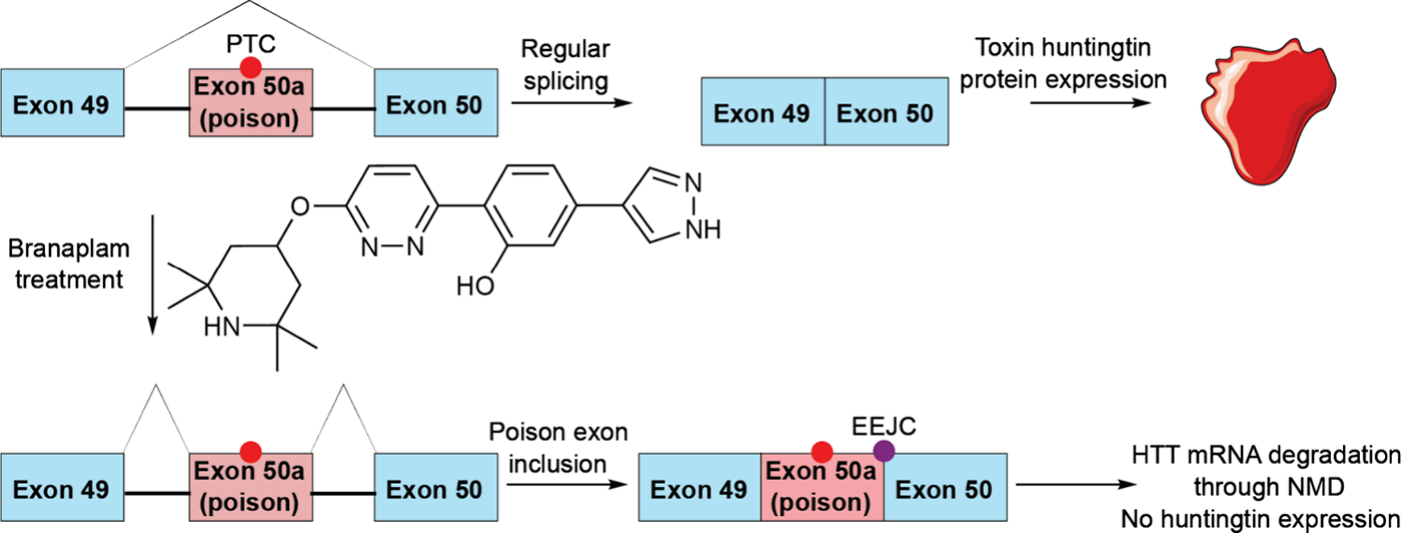
Branaplam induces the degradation of HTT mRNA. It promotes inclusion of a poison exon-containing premature stop codon, which in turn promotes retention of exon–exon junction complexes (EEJC) and recruitment of nonsense-mediated decay (NMD) machinery, leading to degradation of mRNA.

Interestingly, branaplam was also found to affect the mRNA of huntingtin, driver of Huntington’s disease.^324^ It is caused by an expansion of CAG repeats on one of the alleles of this gene.^325^ This results in production of a toxic protein that ultimately causes death of neurons that express it. Complete knockout of huntingtin has been shown to also be neurotoxic, although partial knockdown appears to be well tolerated.^326,327^ Branaplam achieves the latter by forcing inclusion of a premature termination codon (PTC)-containing poison exon (pseudoexon) in huntingtin mRNA (Figure 11).^324^ As was discussed in Section 2.4, mRNAs containing PTC(s) get degraded by RNA quality control machinery, so by forcing poison exon inclusion, branaplam induces degradation of the huntingtin mRNA.

Branaplam was trialed against Huntington’s disease in phase II clinical trials, which culminated in their termination due to the patients suffering nerve damage.^328,329^ This goes in hand with the previous observations that severe reduction in huntingtin levels induces neurotoxicity and branaplam is not allele-specific, e.g., it affects the levels of both the wild type and expansion repeat-containing mRNA. However, branaplam treatment did result in reduction of huntingtin levels in cerebrospinal fluid, which demonstrates that small molecule poison exon splicing modulators can indeed induce RNA degradation in humans. Of note is REM-422, a small molecule which induces poison exon inclusion in the *MYB* oncogene and has a similar mechanism to that of branaplam.^330^ It is developed by Remix Therapeutics and is in clinical trials against adenoid cystic carcinoma and acute myeloid leukemia.

### 3.3 Direct RNA Degrader Small Molecules

As discussed in Section 2.5, the study of RNA stability in various media and ribonuclease mechanisms led to discovery of RNA degrader small molecules. Nonenzymatic RNA degradation can be catalyzed by various metal ions, with the presence of magnesium, zinc, potassium, copper, and other cations leading to faster rates of degradation, through acting as either Lewis acids or radical generators.^331−334^ The presence of certain small molecules in a buffer, including imidazole and morpholine, leads to acceleration of RNA hydrolysis.^335−337^ These observations were key in the development of RNA-degrading small molecules.

**Figure 12:**
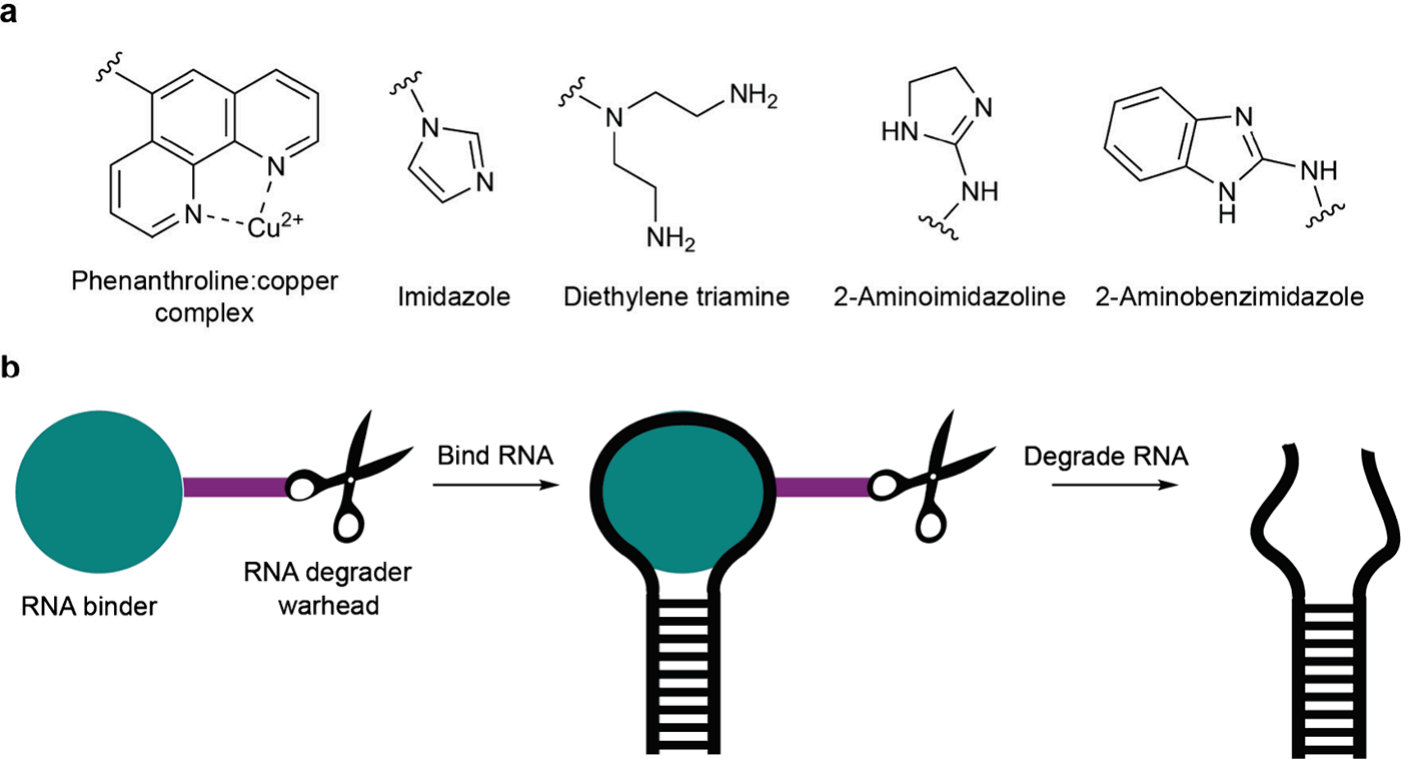
Direct RNA degrader small molecules. (a) Examples of warheads that have been utilized for development of direct RNA degrader small molecules. (b) Mechanism of proximity-induced nucleic acid degraders (PINADs).

The initial RNA degraders were either unguided or antisense-oligonucleotide guided, with the latter already discussed in Section 2.5, and could induce RNA degradation via both transition-metal-dependent and general base mechanisms (Figure 12a). Perhaps the first RNA degrading small molecule to be reported was copper–phenanthroline complex.^338^ The two parts of the complex are responsible for discrete functions—the phenanthroline is a nucleic acid intercalator, whereas copper can generate peroxide and superoxide radicals.^247^ Thus, phenanthroline brings copper to the proximity of a nucleic acid, where it generates radical species, which react with nucleic acids and result in scission of the phosphodiester bond as well as base damage. Copper must be able to cycle between oxidation states +1 and +2 in order to generate radicals thus needs an auxiliary redox agent, such as peroxide, ascorbate, or a thiol.^339^ A number of transition metal complexes have been demonstrated to degrade RNA in a similar manner and were often used as tools to study RNA structures.^340−343^

The bulk of nonmetal-dependent RNA degrader small molecules contain either imidazole (or molecules similar to imidazole) or polyamine warheads. Cyclodextrin–imidazole conjugates reported by Breslow were used to study the imidazole-induced RNA cleavage and inspired further work in this field.^344^ Numerous subsequent degrader molecules consisted of imidazole(s) and amine(s) conjugated to an intercalating moiety, with reported RNA half-lives dependent on concentration of the degrader agent and ranging from one hour to one day.^345−347^ Imidazoline and benzimidazole warheads were also used for this application.^348,349^ Di- and oligoamines were also shown to degrade RNA, with amines simultaneously acting as phosphodiester binders (akin to a positively charged lysine residue acting as a phosphodiester binder in RNase A) and cleavers through acting as bases.^350,351^

In addition to a degrader warhead, a guiding moiety is needed to achieve targeted RNA degradation. As described in Section 2.5, so far the most commonly used strategy was to attach these warheads to an antisense oligonucleotides, an approach with a number of caveats.^236^ Our group found that conjoining a warhead to an RNA-binding small molecule can result in a targeted RNA degrader, and we have dubbed this family of molecules Proximity-Induced Nucleic Acid Degraders (PINADs) (Figure 12b).^352^ To demonstrate the utility of this approach, we have prepared two families of these molecules—one based on the betacoronaviral pseudoknot binder MTDB and another one on a G-quadruplex binder pyridostatin, by attaching them to imidazole via a PEG linker.^353,354^ Both degrader molecules were tested against SARS-CoV-2 infection models and were shown not only to degrade the viral RNA but also to exert an antiviral effect. We have demonstrated target engagement in vitro, showing that degradation occurs when these molecules are incubated with the targeted RNA, as well as in cellular assays, demonstrating that RNA damage accumulates mostly in the targeted locus. Furthermore, these molecules exhibited antiviral effects in cellular and mouse models of SARS-CoV-2 infection. A similar construct was also reported by the Duca group—neomycin, an aminoglycoside RNA binder was conjoined to a histidine which has the RNA degrader imidazole warhead.^355^ These molecules coulddegrade a fragment of HIV Tar RNA in vitro, which provides further evidence for the utility of PINADs as antiviral compounds.

PINADs were demonstrated to have therapeutic potential, but a number of questions remain before this class of compounds can find its way to the clinic. The examples featured so far focus on viral RNA—can PINADs be used for a meaningful reduction of endogenous human RNAs? Perhaps one of the biggest hurdles here is the lack of selective RNA binder small molecules, but recent progress in the field shows that this goal is attainable. RNA turnover rates vary dramatically—can PINADs be utilized for degradation of short-lived substrates or are suitable only for degradation of RNAs with slow turnover rates?^1^ Most of the degrader warhead characterization so far was done in vitro; thus, the field would greatly benefit from the development of systematic cellular assays. If these hurdles are successfully navigated through, PINADs have the potential to succeed where oligonucleotide-based artificial nucleases were found to be lacking.

### 3.4 Direct RNA Degrader Natural Products

**Figure 13:**
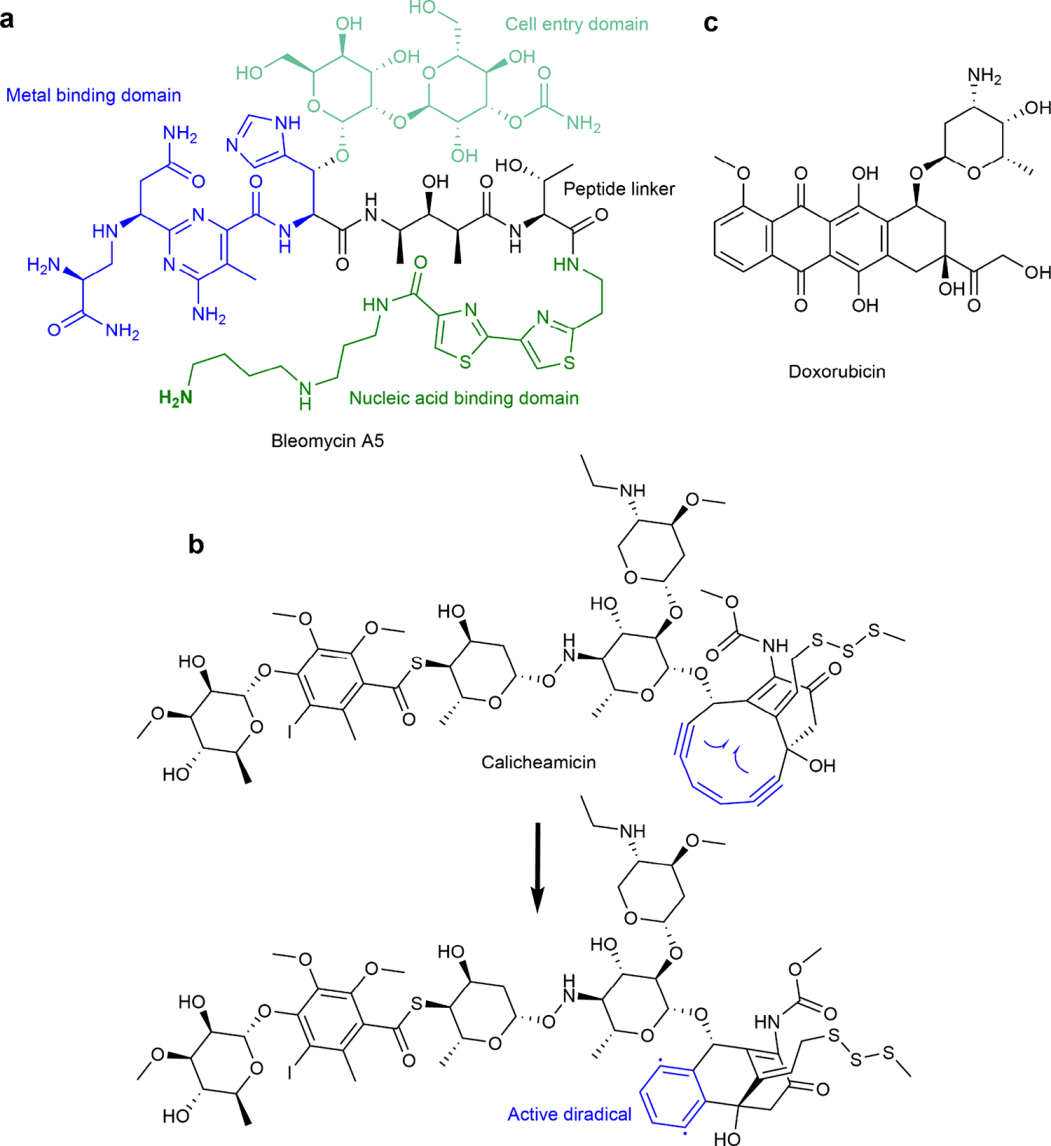
Natural products known to damage nucleic acids. (a) Bleomycins damage DNA and RNA by inducing proximity with transition metals. (b) Enediynes like calicheamicin damage DNA and RNA via radical generation. (c) Anthraquinones like doxorubicin damage DNA by cross-linking it and via radical generation.

Long before the dawn of humanity, nucleic acid degraders were utilized by bacteria in the form of natural products. Some have mechanisms of action akin to ones described previously, but some are distinct. Bleomycins function by bringing nucleic acids to a proximity of a redox-active transition metal such as iron or copper, akin to phenanthrolines (Figure 13a).^356,357^ These rather complex molecules are composed of four domains—the metal binding domain which contains an imidazole in addition to other nitrogen lone pair containing moieties, the nucleic acid binding domain with a bithiazole playing the key role, the carbohydrate domain that facilitates cell targeting, and a peptide-based linker to join the other three.^358−362^ Similarly to copper–phenanthrolines, bleomycins bind nucleic acids and lead to a high local concentration of transition-metal-generated radicals, which are responsible for nucleic acid degradation. For a time, bleomycin was thought only able to cleave DNA but not RNA, but the understanding that this class of degraders is transition-metal-dependent has led to observation that bleomycin–metal complexes can also cleave RNA.^363−366^ Bleomycin is an approved chemotherapy against various cancers, including Hodgkin lymphoma, with the anticancer effect thought to emerge from inhibition of DNA synthesis and, subsequently, cell replication.^367^

Enediynes are another family of nucleic-acid-cleaving natural products. They generate radicals through a different, metal-independent mechanism (Figure 13b).^368^ They contain a large unsaturated system, which under the right conditions will undergo Bergmann rearrangement, resulting in a biradical aromatic ring with the two unpaired atoms in nonbonding sp^2^ orbitals of aromatic carbon atoms. These biradicals abstract hydrogens from the deoxyribose/ribose ring of a nucleic acid, which ultimately results in scission of the phosphodiester bond.^369^ As with bleomycins, enediynes were first demonstrated to cleave DNA, but understanding of their mechanism led to discovery of RNA cleavage.^370^ Like bleomycins, enediynes require nucleic acid binding moieties to induce RNA degradation. For this purpose, calicheamicins possess an oligosaccharide domain, whereas dynemicins contain an anthraquinone moiety, which is not only a nucleic acid intercalator but a redox cycler in itself.^371,372^

Indeed, anthraquinones in the form of anthracyclines are also known to cleave DNA. They contain a large flat aromatic system, which intercalates between the bases of nucleic acids (Figure 12c).^373^ They also contain a redox-cycling anthraquinone moiety, which in the presence of oxygen and a suitable substrate will produce formaldehyde, a nucleic acid alkylating and cross-linking agent.^374^ In the case of DNA, extensive cross-linking will lead topoisomerase 2 poisoning and double strand cleavage, which is the underlying mechanism of anthracycline anticancer activity.^375^ Anthracyclines have also been reported to induce direct oxidative DNA damage in the presence of copper.^376^ They are also known to bind RNA, although no instances of induced degradation have been reported.^377^ Anthracyclines are among the most widely used chemotherapies, as the DNA double strand breaks lead to apoptosis of cancer cells.^378^

In their native forms, RNA-degrading natural products have poor selectivity. To overcome this, Disney and colleagues have conjugated bleomycin A5 onto a binder of CUG repeat, resulting in a molecule that specifically degrades RNAs with a high number of CUGs.^379^ They demonstrated that this construct can reduce the abundance of *DMPK* mRNA containing 500 CUG repeats by up to 40% in a cellular context but has no effect on mRNAs with 17 CUG repeats or less. Similar results were observed in mice harboring 250 CTG-containing *HSA* gene.^380^ They used a similar strategy do design a bleomycin-degrader of pre-miR-372, which has the preferred bleomycin cleavage site.^381^ These results highlight that RNA-cleaving natural products can serve as warheads in targeted RNA degraders, although their relatively high molecular weight and complex synthetic pathways pose design challenges. Notably, bleomycin has also been conjugated to a guide oligonucleotide but to induce DNA rather than RNA degradation, but the construct was found to induce autodegradation, limiting the applicability of this approach.^382^

### 3.5 RNA Structure Disrupts As Stability Modulators

**Figure 14:**
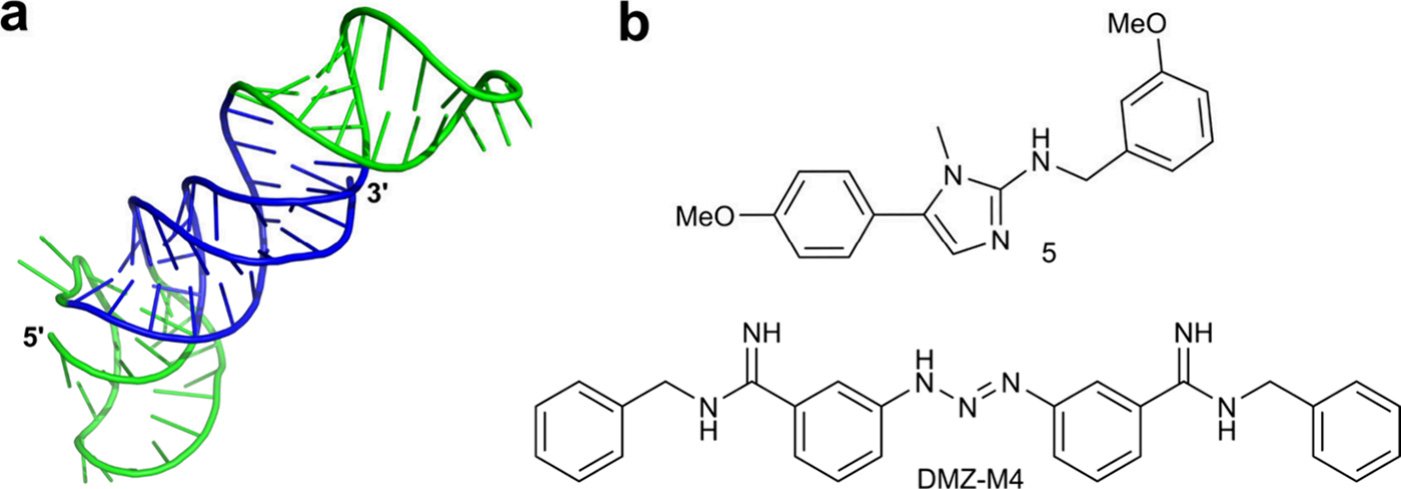
MALAT1 triple helix can be destabilized by small molecules. (a) Crystal structure of MALAT1 triple helix (PDB entry 4PLX).^385^ (b) Examples of small molecules demonstrated to destabilize MALAT1 triple helix and reduce its half-life in cells or stability toward nucleases.

RNA structure and turnover rates are tightly interconnected. This phenomenon is exploited by viruses—some viral RNAs contain a U-rich expression and nuclear retention element (ENE).^383^ It can hybridize with the poly(A) tail of the transcript, which protects it from deadenylation and thus extend its half-life.^384^ A long noncoding RNA *MALAT1* also contains an ENE-like structural element, which forms a triple helix (Figure 14a).^385^ Weakening of this triple helix by mutating one of its nucleotides was demonstrated to reduce the half-life of this structural element 3-fold. This triple helix has been proposed to protect *MALAT1* from 3′-exonucleases.^386^ As such, its destabilization leads to an increased rate of transcript decay, which is of therapeutic interest as *MALAT1* has been implied to be relevant for cancer metastasis.^387^

Small molecules can also destabilize this structural element and facilitate *MALAT1* decay (Figure 14b). Abulwerdi et al. report molecules which both bind and destabilize the triple helix of *MALAT1*, leading to decreased levels of the transcript.^388^ The binders were identified through a small molecule microarray approach, which led to discovery of 28 commercially available small molecules that interact with *MALAT1* triple helix.^296^ These compounds were then used to treat an organoid model of mammary cancer, followed by measurement of the *MALAT1* levels via *qPCR*. Two of the compounds were found to reduce *MALAT1* levels by >40%, one of which was found to destabilize the triple helix, this likely being the mechanism behind the transcript’s depletion. Two further studies by the Hargrove group reported that small molecule binders of MALAT1 triple helix can alter the transcript’s resistance toward exonucleases.^389,390^ Notably, one of the two studies discovered both structure-stabilizing and structure-destabilizing molecules, with the former leading to protection from RNase A degradation and the latter having an opposite effect. Altogether, these findings demonstrate that a binder of suitable RNA structure can be sufficient to reduce its levels through modulation of the structure’s stability. The growing wealth of knowledge about RNA structures can guide the discovery of new molecules with this mechanism of action against targets of therapeutic interest.

## 4 Uses Cases, Limitations, and Perspective for Targeted RNA Degraders

### 4.1 Applications and Limitations of Nucleic-Acid-Guided Methods

Nucleic-acid-based degraders are already having a huge impact on healthcare, enabling the development of drugs against targets unapproachable via traditional medicinal chemistry approaches. The ability to deplete targeted RNA makes these approaches exceptionally suitedagainst gain-of-function diseases. As nucleic acid therapeutics target sequences, they are ideal against long RNA species with high variability in their nucleic acid code, in particular mRNAs, but targeting of short and sequentially similar RNA species, such as miRNAs and tRNAs, remains challenging.^391^ Furthermore, siRNAs do not efficiently target nuclear transcripts; thus, for this modality, cellular transcript localization can also be an issue.^18,392^ Conversely, both RNase H and Cas13 were shown to be proficient in nuclear transcript degradation. Perhaps the most severe limitations of nucleic-acid-based therapeutics arise from delivery and tissue targeting challenges. In general, oral bioavailability of oligonucleotides is poor, and although it is not impossible to increase it to acceptable levels, all currently approved oligonucleotide therapeutics are delivered through alternative routes of administration.^393,394^ So far, the most successful antisense oligonucleotides target the liver, with delivery to other tissues still posing a great challenge. Furthermore, nucleic-acid-based agents require delivery vectors or ligands, which are an additional source of adverse effects and require tuning for optimal pharmacokinetics.^395,396^ As siRNA and RNase H-inducing oligonucleotide efficacy is well established, perhaps the biggest opportunity for the field lies in targeting other tissues. Altogether, these limitations warrant the need for different approaches to targeted RNA degradation, and most of them can be overcome via RNA-degrading small molecules.

### 4.2 Perspective for Small Molecule Targeted RNA Degraders

Although RNA-degrading small molecules have been known for decades, only in recent years have scientists started envisioning nonoligonucleotide constructs which both target and degrade RNA. A number of new RNA degrader technologies were recently reported, and coupled with the ongoing boom of RNA-targeting small molecule development, they hold the promise to greatly expand the space of druggable biomolecules, with a superior tissue accessibility compared to oligonucleotide-based methods. Still, hurdles besides RNA ligand discovery remain. Many of the reported technologies result in substrates with a large molecular weight—how to best circumvent this? We can look for answers in the field of targeted protein degradation, where the relatively large size of PROTACs did not prohibit them from entering the clinic but has posed pharmacokinetic challenges.^397^ Many small molecule methods to date result in far from complete RNA degradation, with a ∼50% decrease often being the limit—is this an inherent limitation of these methods? This phenomenon poses another challenge—as the extent of degradation with small molecule degraders is often much lower than that of siRNA, it can be difficult to pick it up via standard methods such as qPCR due to a lack of sensitivity. This, together with the fact that in vitro degradation rates depend on the buffer, leads to reproducibility issues. As such, the field would benefit from more accurate and standardized assays. Another hurdle for small molecule degraders is their orientation in space. Most of the described molecules herein are bifunctional, and thus, their activity depends on their spatial orientation; e.g., RIBOTAC must be able to simultaneously bind RNA and RNase L. As such, rational spatial design is not possible without knowing the absolute RNA structure. This, together with limited knowledge about the role of linkers, complicates the design of bifunctional degraders for new RNA targets.

Targeting specificity of RNA binders is another concern, but as we know from studies of kinase inhibitors, a nonspecific binder can still be a clinically useful pharmaceutical. Furthermore, it has been demonstrated that RNA binders are not necessarily less selective than protein binders.^398^ As such, small molecule targeted RNA degraders have the potential to become a prime strategy to target biomolecules undruggable on a protein level. With the resolution of the aforementioned hurdles, the field will be able to pursue at least two high value directions—targeting mRNAs of undruggable proteins and targeting noncoding RNAs, with greater tissue accessibility and easier administration than nucleic-acid-based methods.

### 4.3 Targeted RNA Degradation and Expansion of the Druggable Biomolecule Space

Nucleic-acid-guided targeted RNA degraders have already demonstrated that if a disease has no associated druggable protein target, then drugging the mRNA can be a valid strategy instead. Coding RNAs form only the tip of the iceberg—most of the human genome is transcribed as noncoding RNAs. Understanding of their underlying biology still lags behind that of coding RNAs, but their relevance to human disease is established.^399−401^ Novel tools to study the link between noncoding RNAs and pathology could cement these transcripts as druggable targets, and targeted RNA degraders will likely have a role to play here. As such, it is difficult to think of another approach that could expand the space of druggable biomolecules as much as targeting and modulating RNA. The past two decades have seen tremendous progress in oligonucleotide-based targeting methods, which have been utilized mostly to treat various liver pathologies. The advent of small molecule approaches may well result in novel medicines against diseases in a broad range of tissues. For both targeting moieties, two paths of development are open. Degraders that recruit ribonucleases exploit their incredible efficiency, but due to uneven expression and localization patterns, such degraders are context dependent. Direct degraders exhibit slower RNA turnover rates but do not rely on biomolecular machinery. Altogether, the expansion of druggable biomolecule space through targeted RNA degradation has the potential to completely change the perception of what is a druggable target.
